# Inter‐subject stability and regional concentration estimates of 3D‐FID‐MRSI in the human brain at 7 T

**DOI:** 10.1002/nbm.4596

**Published:** 2021-08-11

**Authors:** Gilbert Hangel, Benjamin Spurny‐Dworak, Philipp Lazen, Cornelius Cadrien, Sukrit Sharma, Lukas Hingerl, Eva Hečková, Bernhard Strasser, Stanislav Motyka, Alexandra Lipka, Stephan Gruber, Christoph Brandner, Rupert Lanzenberger, Karl Rössler, Siegfried Trattnig, Wolfgang Bogner

**Affiliations:** ^1^ High‐field MR Center, Department of Biomedical Imaging and Image‐guided Therapy Medical University of Vienna Vienna Austria; ^2^ Department of Neurosurgery Medical University of Vienna Vienna Austria; ^3^ Division of General Psychiatry, Department of Psychiatry and Psychotherapy Medical University of Vienna Vienna Austria; ^4^ Institute for Clinical Molecular MRI Karl Landsteiner Society St. Pölten Austria; ^5^ High‐field MR Center, Center for Medical Physics and Biomedical Engineering Medical University of Vienna Vienna Austria

**Keywords:** 7 T, healthy brain, high resolution, inter‐subject reproducibility, MRS, MRSI

## Abstract

**Purpose:**

Recently, a 3D‐concentric ring trajectory (CRT)‐based free induction decay (FID)‐MRSI sequence was introduced for fast high‐resolution metabolic imaging at 7 T. This technique provides metabolic ratio maps of almost the entire brain within clinically feasible scan times, but its robustness has not yet been thoroughly investigated. Therefore, we have assessed quantitative concentration estimates and their variability in healthy volunteers using this approach.

**Methods:**

We acquired whole‐brain 3D‐CRT‐FID‐MRSI at 7 T in 15 min with 3.4 mm nominal isometric resolution in 24 volunteers (12 male, 12 female, mean age 27 ± 6 years). Concentration estimate maps were calculated for 15 metabolites using internal water referencing and evaluated in 55 different regions of interest (ROIs) in the brain. Data quality, mean metabolite concentrations, and their inter‐subject coefficients of variation (CVs) were compared for all ROIs.

**Results:**

Of 24 datasets, one was excluded due to motion artifacts. The concentrations of total choline, total creatine, glutamate, myo‐inositol, and *N*‐acetylaspartate in 44 regions were estimated within quality thresholds. Inter‐subject CVs (mean over 44 ROIs/minimum/maximum) were 9%/5%/19% for total choline, 10%/6%/20% for total creatine, 11%/7%/24% for glutamate, 10%/6%/19% for myo‐inositol, and 9%/6%/19% for *N*‐acetylaspartate.

**Discussion:**

We defined the performance of 3D‐CRT‐based FID‐MRSI for metabolite concentration estimate mapping, showing which metabolites could be robustly quantified in which ROIs with which inter‐subject CVs expected. However, the basal brain regions and lesser‐signal metabolites in particular remain as a challenge due susceptibility effects from the proximity to nasal and auditory cavities. Further improvement in quantification and the mitigation of *B*_0_/*B*_1_‐field inhomogeneities will be necessary to achieve reliable whole‐brain coverage.

## Graphical abstract

We have assessed the typical amplitude and variability of quantitative concentration estimates of 3D‐CRT‐FID‐MRSI at 7 T in healthy volunteers. We successfully estimated, using an internal water reference, metabolite concentrations in 44 brain ROIs that agree with previous literature and determined inter‐subject coefficients of variation. Our results are a step towards future metabolic high‐resolution atlases of the human brain.

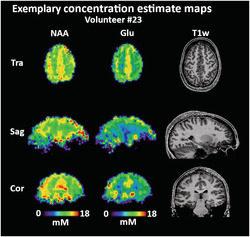

**Abbreviations**
Asp: aspartate
CRLB: Cramér‐Rao lower bound
CRT: concentric ring trajectory
CSF: cerebrospinal fluid
EPSI: echo‐planar spectroscopic imaging
FID: free induction decay
FWHM: full width at half maximum
GABA: γ‐aminobutyric acid
Gln: glutamine
Glu: glutamate
Gly: glycine
GM: gray matter
GSH: glutathione
mIns: myo‐inositol
MRSI: magnetic resonance spectroscopic imaging
NAA: *N*‐acetylaspartate
NAAG: *N*‐acetylaspartylglutamate
ROI: region of interest
SAR: specific absorption rate
Ser: serine
sIns: scyllo‐inositol
SNR: signal‐to‐noise ratio
SVS: single‐voxel spectroscopy
Tau: taurine
tCho: phosphocholine + glycerophosphocholine
tCr: total creatine (creatine + phosphocreatine)
*T*_E_
: echo time
*T*_R_
: repetition time
WET: water suppression enhanced through *T*_1_ effects
WM: white matter

## 1 INTRODUCTION

Proton magnetic resonance spectroscopic imaging (MRSI) in the brain based on the direct acquisition of the free induction decay (FID) signal has been introduced to overcome many technical challenges with ultra‐high‐field systems.^1^, ^2^ Such challenges include lower *B*_0_/*B*_1_^+^ homogeneity, more restricted specific absorption rate (SAR), and shorter *T*_2_ times.^3^ This simple acquisition scheme further reduces SAR, eliminates signal loss due to *T*_2_ relaxation and *J* coupling, improves spatial selection, and enables short repetition times (*T*_R_).^1^, ^2^ Over the last several years, technical improvements have concentrated on faster data acquisition to reach shorter measurement times, increased brain coverage, and higher spatial resolutions, which are attractive for clinical metabolic brain mapping.^4^ Starting with parallel imaging techniques,^5^, ^6^, ^7^, ^8^ echo‐planar spectroscopic imaging (EPSI),^9^ and *T*_R_ reduction,^10^, ^11^, ^12^ these recent innovations have concentrated on spatial‐spectral encoding using spiral,^13^, ^14^ rosette,^15^ and concentric ring trajectories (CRTs).^16^, ^17^, ^18^, ^19^, ^20^ Research culminated in a CRT‐based 3D‐MRSI sequence at 7 T that can cover the whole brain with ~3 mm isotropic resolution, acquired in 10‐15 min.^21^, ^22^

7 T MRSI, with an increased signal‐to‐noise ratio (SNR) and spectral dispersion, compared with lower‐field MR scanners, enables imaging of a wide range of metabolites, eg improving the separation of *N*‐acetylaspartate (NAA) from *N*‐acetylaspartylglutamate (NAAG), glutamate (Glu) from glutamine (Gln), or glycine (Gly) from myo‐inositol (mIns).^22^, ^23^, ^24^ Based on these benefits, 7 T MRSI has been successfully applied to research applications ranging from γ‐aminobutyric acid (GABA) mapping^25^, ^26^ to the resolution of metabolism in tumors,^9^, ^22^, ^23^, ^24^, ^27^ multiple sclerosis,^28^ and epilepsy.^29^

While many technical milestones have been reached, open questions remain with respect to the stability of these whole‐brain and/or high‐resolution MRSI methods. This includes inter‐subject variations of metabolite concentrations in different brain regions, as well as intra‐subject variations over time. So far, we had only investigated these variations for FID‐MRSI in a small study at 3 T.^30^ Historically, the results of MRSI quantification have varied greatly depending on study design, data evaluation, and investigated brain regions.^31^, ^32^, ^33^, ^34^, ^35^, ^36^, ^37^ To facilitate the use of MRSI for clinical and research applications, we have to first establish the normal concentrations of metabolites within different brain regions of healthy volunteers instead of relative signals as previously.

To evaluate the performance and inter‐subject stability of our 7 T 3D‐CRT‐FID‐MRSI approach, we conducted a study with a larger subject cohort, detailed quantification estimation and regional evaluation.

### 1.1 Purpose

The purpose was to acquire whole‐brain, 3D‐CRT‐based FID‐MRSI at 7 T in a wider cohort (24 volunteers) than in our previous volunteer studies and to derive for the first time concentration estimates for our method, and further to assess the local MRSI data quality in an array of different small and large brain regions in order to evaluate the quantification robustness and inter‐subject variability for the concentration estimates of individual metabolites.

The results will define the performance limits of our 3D‐CRT‐based FID‐MRSI method at 7 T in regard to which metabolites can be confidently and reliably mapped in which regions of the brain.

## 2 EXPERIMENTAL

### 2.1 Subject recruitment

This study was conducted with the approval of the local institutional review board. Subjects were included in this study when no contraindications for 7 T MRI (eg claustrophobia, ferromagnetic implants, non‐ferromagnetic metal head implants >12 mm, or pregnancy) were reported. Written and informed consent was obtained from all 24 young healthy volunteers (12 male, 12 female, mean age 27 ± 6 years, Table 1). We chose a young cohort due to expected good compliance for motionlessness and easy reproducibility.

**TABLE 1:** Overview of all volunteer subjects measured in this study. In some volunteers, individual ROIs had to be excluded due to poor segmentation of the GM from the WM

Volunteer	Sex	Age [years]	Regions excluded due to poor GM/WM segmentation
1	Male	24	
2	Male	34	
3	Male	23	
4	Male	23	
5	Female	33	Parietal GM, WM, GM + WM
6	Female	23	
7	Female	24	
8	Female	29	Parietal GM, WM, GM + WM
9	Male	33	Frontal, motor, parietal GM, WM, GM + WM
10	Female	19	
11	Female	21	
12	Female	20	
13	Male	31	
14	Male	23	
15	Male	38	
16	Male	27	
17	Male	35	
18	Male	39	
19	Female	22	
20	Female	23	Parietal GM, WM, GM + WM
21	Female	24	
22	Male	34	
23	Female	23	
24	Female	20	Frontal, motor, parietal GM, WM, GM + WM

### 2.2 7 T MRSI measurement protocol

We performed the measurement protocol using a 7 T whole‐body MR imager (Magnetom, Siemens Healthineers, Erlangen, Germany), located at the High Field MR Centre of the Medical University of Vienna, featuring a gradient system with a 70 mT/m maximum gradient strength per direction and a 200 mT/m/s slew rate as well as a 32‐channel head receive coil array (Nova Medical, Wilmington, MA). The protocol included *B*_1_^+^ maps for flip‐angle optimization, *B*_0_ maps, and magnetization prepared two rapid gradient echoes (MP2RAGE) as the *T*_1_‐weighted morphological MRI reference (*T*_R_ 5000 ms, *T*_E_ 4.13 ms, *T*_I1_ 700 ms, *T*_I2_ 2700 ms, 0.75 × 0.75 × 0.75 mm^3^ resolution, 8 min 2 s with GRAPPA factor 3).

Our 3D‐CRT‐FID‐MRSI sequence (a detailed description of sequence design and implementation can be found in Reference^21^) employed in‐plane 2D‐CRT, and through‐plane phase encoding of an ellipsoidal *k*‐space resulted in a 64 × 64 × 39 matrix over a field of view of 220 × 220 × 133 mm^3^. This corresponds to a nominal spatial resolution of 3.4 × 3.4 × 3.4 mm^3^ and an effective resolution of 4.7 × 4.7 × 4.7 mm^3^ or 0.1 cm^3^. A slab of 110 mm thickness starting at the superior part the brain was selected with slices oriented in parallel to the horns of the corpus callosum. Other MRSI scan parameters included *T*_R_ of 450 ms, scan time of 15 min, acquisition delay of 1.3 ms, 39°excitation flip angle (calculated as nominal average Ernst angle of NAA, tCr (creatine + phosphocreatine), tCho (phosphocholine + glycerophosphocholine), Glu, mIns^5^), readout duration of 345 ms, spectral bandwidth of 2778 Hz, variable temporal interleaves (eg 1‐3 depending on the respective ring radii to maintain the spectral bandwidth for larger readout circles), and 7 T‐optimized WET water suppression.^10^, ^38^ No lipid suppression was employed during acquisition to allow a short *T*_R_. A second MRSI scan, without water suppression, included a *T*_R_ of 200 ms, a readout duration/spectral bandwidth of 158 ms/606 Hz, and an Ernst angle of 27°, but with otherwise identical spatial coverage, and was acquired in 3 min 18 s as an internal water reference. This second scan was necessary as we required an unsuppressed water signal as reference.

### 2.3 Data processing and quantification

For offline MRSI processing, we utilized our in‐house‐developed software pipeline^39^ that is based on MATLAB (MathWorks, Natick, MA), Bash (Free Software Foundation, Boston, MA), FSL (Analysis Group, FMRIB, Oxford, UK), and MINC (MINC Tools, McConnell Brain Imaging Center, Montreal, QC, Canada). This pipeline included iMUSICAL (interleaved multichannel spectroscopic data combined by matching image calibration data) coil combination based on interleaved water calibration scans,^17^, ^20^ *k*‐space reconstruction with in‐plane convolution gridding^20^, ^40^ (weighting non‐Cartesian points in relation to the Cartesian target point), off‐resonance correction to compensate the time delay of acquisition samples^41^ and spatial Hamming filtering, as well as post‐measurement lipid signal removal by L2 regularization^42^, ^43^ prior to spectral quantification. Reconstruction did not include eddy current correction, as CRTs are inherently resistant to these.^18^ It further did not include *B*_0_ or *B*_1_ correction (while differences in local flip angles can lead to differences in the effective *T*_1_ weighting, we previously found only little impact for FID‐MRSI^44^). A graphical overview of the reconstruction pipeline is available in Supporting Figure 1.

Each spectrum was separately quantified via LCModel (v6.3‐1, LCMODEL, Oakville, Ontario, Canada)^45^ over evaluation ranges of 0.2‐1.2 ppm and 1.8‐3.88 ppm (excluding the lipid spectral range of 1.2‐1.8 ppm due to possible remaining lipid signal after L2 regularization; the upper limit of 3.88 was necessary due to water suppression effects). Our basis set, simulated in NMRscope‐B^46^ accounting for the first‐order phase caused by the acquisition delay of 1.3 ms, included the following neurochemicals: tCr, tCho, NAA, NAAG, Glu, Gln, mIns, scyllo‐inositol (sIns), GABA, glutathione (GSH), Gly, taurine (Tau), cysteine, serine (Ser), and aspartate (Asp). An average macromolecular background based on prior studies with metabolite‐nulled measurements was included to improve quantification.^47^, ^48^ Water was quantified separately from the unsuppressed reference scan, using LCModel as well, with a water basis simulated as above. SNR (using the pseudo‐replica method with receiver noise prescans acquired at the start of the MRSI sequence^6^) and full width at half maximum (FWHM) were calculated voxel‐wise from the LCModel fits of NAA and tCr at 3.02 ppm.

For the calculation of concentration estimates using the internal water concentration as a reference, *T*_1_‐weighted images were segmented into gray matter (GM)/white matter (WM)/cerebrospinal fluid (CSF) using FSL's FAST tool and the segmentations were resampled to the MRSI resolution. Only the GM/WM segmentations were used for further analysis. Molar concentrations of 36.1 moL/L for GM, 43.3 moL/L for WM, and 53.8 moL/L for CSF were assumed based on literature.^49^ The *T*_1_ relaxation times, which are necessary for the correction of saturation effects at short *T*_R_, were taken from the literature or estimated as an average of known *T*_1_ values for metabolites without published human 7 T values (Table 2).^50^, ^51^ Due to the use of an ultra‐short acquisition delay of 1.3 ms, we did not deem *T*_2_ corrections necessary. Metabolite concentrations were estimated as previously described^52^, ^53^: in short, for every MRSI voxel, tissue segmentation was used to calculate *T*_1_ correction factors for all metabolites and water, according to the GM and WM content, and applied to the signal derived from the LCModel fitting results. Concentration estimates for each spectrum were then calculated as the ratio of metabolite‐to‐water signal multiplied by the local water concentration and the correction factor calculated before influenced by the respective GM/WM/CSF fraction as well as *T*_1_ relaxation.

**TABLE 2:** *T*_1_ times for metabolites and water for GM and WM used for concentration estimates in this study. Lacking reported literature values for human in vivo MRS at 7 T, we used an average of known 7 T *T*_1_ times (ie NAA, tCr, tCho, mIns, Glu, Gln, GSH, Tau, NAAG) for Gly and Ser

Compound	*T*_1_ GM [ms]	*T*_1_ WM [ms]	Reference
Asp	1000	1000	Estimated from^51^
tCho	1510	1320	^50^
tCr 3 ppm	1780	1740	^50^
GABA	1100	1200	^94^
Glu	1610	1750	^50^
Gln	1540	1740	^50^
Gly	1400	1400	Average estimate from known compounds
GSH	1140	1060	^50^
mIns	1280	1190	^50^
NAA	1535	1545	,^50^ average of resonances
NAAG	1210	940	^50^
Ser	1400	1400	Average estimate from known compounds
Tau	2150	2090	^50^
Water	2000	1550	^50^

We created 3D maps of concentration estimates for all metabolites and filtered these based on a spectral quality mask, motivated by recent consensus recommendations,^54^ which excluded voxels with at least one of these parameter restrictions: tCr SNR < 5, tCr FWHM > 0.15 ppm, metabolite Cramér‐Rao lower bound (CRLB) > 40%, and metabolite fit value > 13 median absolute deviations. For display purposes, these maps were interpolated tri‐linearly to fourfold resolution in MINC's register tool.

FreeSurfer (6.0, Laboratory for Computational Neuroimaging at the Athinoula A. Martinos Center for Biomedical Imaging, Boston, MA)^55^, ^56^ was used for automated segmentation of structural images based on cortical and subcortical brain atlases.^56^, ^57^ In‐house MATLAB codes were used for mask extraction within each region of interest (ROI). We defined 55 ROIs, as shown in Table 3, including small and large structures/cortices, GM and WM regions separated and merged, and ROIs per hemisphere, in order to investigate which brain regions and ROI sizes could be reliably imaged. Metabolic maps were interpolated to the 0.8 mm resolution of structural images using nearest‐neighbor interpolation. Interpolated maps were overlaid with derived masks and mean metabolite concentrations were calculated within each ROI.^58^

**TABLE 3:** Evaluation of region fitting quality, including rejected regions. ROIs were separated based on the percentage of voxels that fulfilled the criteria of all of NAA, tCr, tCho, and mIns with CRLBs < 40%. Further listed is the estimated GM/WM content of our segmentations, mean ROI size (for comparison, effective voxel size = 0.1 cm^3^) as well as the percentage of voxels for a metabolite in an ROI that had a CRLB of <20% for NAA, tCr, tCho, and Ins, and <40% for all others

*ROI > 80%*	GM [%]	WM [%]	Mean ROI size [cm^3^]	tCho [%]	tCr [%]	GABA [%]	Glu [%]	Gln [%]	Gly [%]	GSH [%]	mIns [%]	NAA [%]	NAAG [%]	Ser [%]	Tau [%]
Subcortical WM (left)	35	63	101.4	85	81	47	83	50	33	46	78	84	59	61	52
Subcortical WM (right)	35	62	101.0	87	84	50	85	52	34	50	82	85	62	62	57
Subcortical WM (bilateral)	35	63	202.4	86	82	48	84	51	33	48	80	85	61	61	54
Motor subcortex WM	31	68	18.4	98	97	74	97	50	54	54	97	98	90	78	78
Motor cortex GM	63	27	18.7	86	83	62	86	55	36	32	81	87	70	67	70
Motor cortex/subcortex GM+WM	47	47	37.1	92	90	68	92	53	45	43	89	92	80	72	74
Parietal subcortex WM	35	64	47.6	98	97	64	96	60	54	58	96	98	84	78	77
Parietal cortex GM	63	27	66.0	91	89	62	91	66	45	42	87	92	72	71	76
Parietal cortex/subcortex GM + WM	50	44	103.6	94	93	63	93	63	49	49	91	95	77	74	77
Cingulate subcortex WM	31	69	13.8	91	87	53	88	37	26	64	84	91	64	61	40
Cingulate cortex GM	77	23	11.0	91	89	66	92	60	29	52	85	91	68	68	62
Cingulate cortex/subcortex GM + WM	51	49	24.9	91	88	59	90	47	27	58	84	91	66	64	50
Visual subcortex WM	33	60	13.6	90	82	29	84	39	33	40	74	89	49	71	56
Primary somatosensory subcortex WM	39	57	7.2	95	93	64	95	61	41	40	93	95	80	74	72
Primary somatosensory cortex/subcortex GM + WM	49	41	16.8	87	84	57	87	57	36	32	83	88	69	66	67
Thalamus	42	58	7.4	90	84	49	84	37	14	51	75	87	58	53	27
Putamen	55	45	5.1	89	86	39	90	67	11	53	72	87	46	54	32
Non‐lobe WM	10	90	31.9	94	91	45	86	34	31	73	86	93	68	61	36
*ROI > 66%*															
Cortical GM (left)	65	26	124.3	69	63	40	69	48	23	30	59	69	44	50	47
Cortical GM (right)	61	25	123.9	78	74	46	77	55	28	36	71	77	52	55	57
Cortical GM (bilateral)	63	25	248.2	73	69	43	73	52	25	33	65	73	48	52	52
Cortical GM + subcortical WM (left)	51	43	225.7	76	71	43	75	49	27	37	68	76	51	55	49
Cortical GM + subcortical WM (right)	49	43	224.9	82	78	48	81	54	30	42	76	81	57	58	57
Cortical GM + subcortical WM (bilateral)	50	43	443.8	79	75	46	78	51	29	40	72	78	54	56	53
Subcortical GM (left)	51	48	14.8	83	78	35	78	43	11	52	65	78	44	42	25
Subcortical GM (right)	50	50	14.4	77	68	32	68	40	9	45	57	69	32	39	19
Subcortical GM (bilateral)	51	49	29.3	80	73	33	72	41	10	49	61	74	38	40	22
Auditory subcortex WM	43	53	7.0	85	79	39	81	62	19	41	75	79	42	55	42
Auditory cortex GM	62	24	12.2	73	67	35	70	55	15	31	60	68	33	45	41
Auditory cortex/subcortex GM + WM	54	36	19.2	77	71	36	74	58	16	35	66	72	36	49	41
Occipital subcortex WM	34	61	22.4	86	79	30	80	40	30	39	73	84	45	64	51
Occipital cortex GM	59	28	29.5	75	66	30	73	41	22	28	60	74	40	56	49
Occipital cortex/subcortex GM + WM	48	43	51.9	80	71	30	76	41	26	33	65	78	42	59	50
Temporal subcortex WM	38	59	33.8	72	67	33	70	50	19	39	64	69	43	47	35
Temporal cortex/subcortex GM + WM	52	41	77.0	66	60	32	64	49	17	33	56	62	38	44	35
Frontal subcortex WM	34	64	80.8	82	80	50	83	51	28	46	77	82	57	55	50
Frontal cortex GM	62	23	96.4	66	62	41	68	48	17	31	59	67	41	43	44
Frontal cortex/subcortex GM + WM	49	43	177.2	73	70	45	74	50	22	38	67	74	48	48	47
Visual cortex GM	55	28	17.1	76	65	26	76	39	22	27	58	79	42	61	49
Visual cortex/subcortex GM + WM	45	43	30.7	82	73	27	80	39	27	33	65	84	45	65	52
Primary somatosensory cortex GM	57	28	9.6	80	77	52	81	55	32	26	75	82	61	60	63
Pallidum	13	87	1.9	81	79	37	75	43	4	53	55	76	32	35	13
Hippocampus	72	26	4.1	81	69	23	65	37	12	51	56	66	27	28	23
Corpus callosum	29	71	1.8	83	73	41	77	25	17	58	68	82	49	48	19
Mean	47	47	67.0	83	78	45	80	49	27	43	73	81	54	57	49

Below quality threshold: brain stem, cerebellum (left, right, bilateral), cerebral WM (left, right, bilateral), amygdala, nucleus accumbens, temporal cortex (GM), ventral diencephalon.

### 2.4 Data evaluation

#### 2.4.1 General overview of measurement quality

Metabolite maps of all subjects were controlled by a reader (G.H.) for the presence of lipid and movement artifacts. Quantification of all metabolites listed in the basis set was evaluated and metabolites that were not fit in at least 10% of brain voxels were discarded from further evaluation. Cysteine was discarded as well due to general doubts about its quantification. We compared concentration estimate maps with uncorrected metabolite maps for differences in data quality and contrast. Representative spectra and metabolite maps were selected for display.

#### 2.4.2 Quantification quality within ROIs

##### Regions

Region‐specific data quality was assessed by calculating the percentage of voxels within an ROI that had CRLBs less than 40% for all of NAA, tCr, tCho, and mIns. ROIs with more than 80% of voxels above that threshold were defined as good and those with 66‐79% as acceptable, and those with less than 60% were rejected. Rejected ROIs were excluded from further evaluation. To also quantify the performance of individual metabolite fitting, the percentage of voxels with CRLBs less than 20% for NAA, tCr, tCho, Ins, and CRLBs less than 40% for all others were determined for every ROI.

#### 2.4.3 Metabolites

Only metabolites that were fit in more than 66% of voxels (mean of all regions) were considered as qualified for the main analysis, but the remaining ones are included in the Supporting Information (Supporting Tables 3 and 4).

#### 2.4.4 Quantification estimates

For all metabolites in all qualified ROIs, regional means per subject and inter‐subject mean of means for all subjects not excluded in Section 2.4.1 were calculated. The range of observed ROI concentration estimates was compared with literature values. To facilitate the comparison with other studies that used ratios to tCr, we additionally calculated metabolite ratios to tCr.

#### 2.4.5 Inter‐subject coefficients of variation

As a measure for the expected variability in metabolite estimates based on physiologic differences among subjects and the stability of our MRSI method, inter‐subject coefficients of variation (CVs) of mean ROI‐specific concentration estimates and ratios to tCr were calculated and compared for all qualified ROIs and metabolites based on the mean concentration estimates/ratios per subject for every ROI. This mean and its standard deviation were then used for CV calculation per ROI over all subjects.

## 3 RESULTS

### 3.1 General overview of measurement quality

**FIGURE 1:**
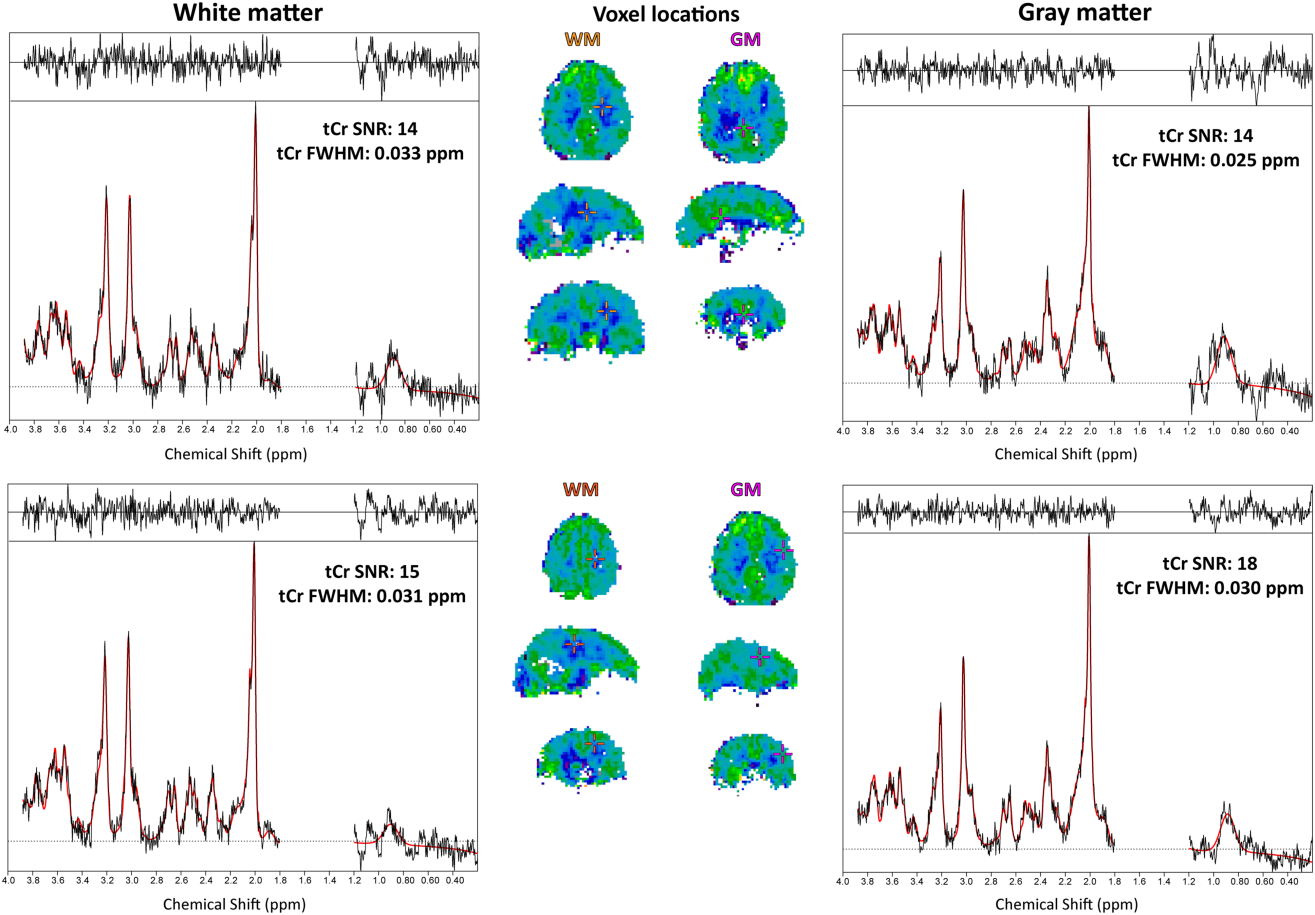
Sample spectra for pure GM and WM voxels in Volunteer 11. The spectra were first‐order phased for viewing convenience. GM/WM differences are especially visible for Glu. Spectral phasing is due to the 1.3 ms acquisition delay. In these examples, only small residual lipid signals remain visible

**FIGURE 2:**
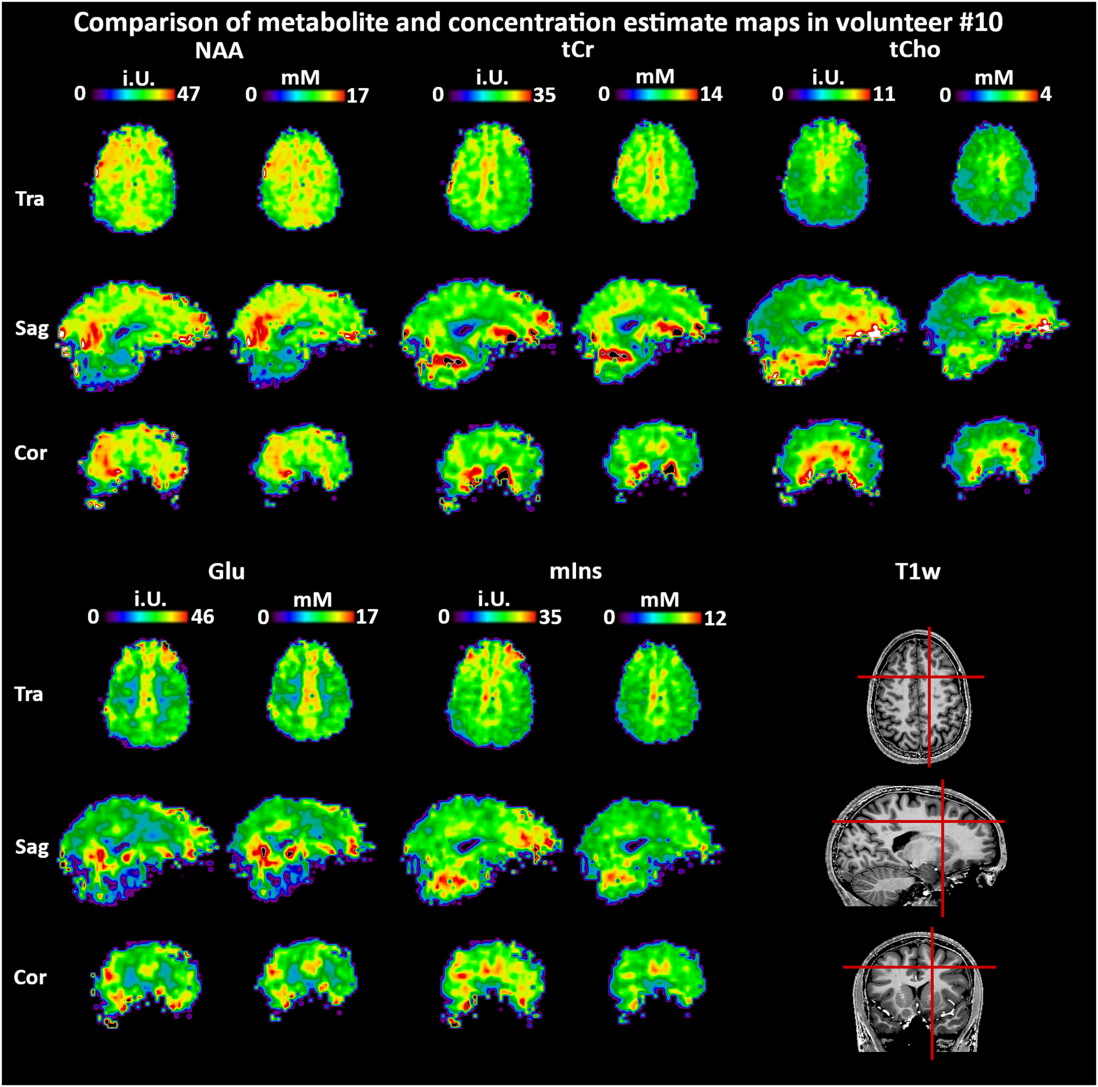
Comparison of metabolite maps prior to water referencing and *T*_1_ corrections with concentration estimate maps in one volunteer for the main metabolites. *T*_1_ corrections and water referencing appear to reduce regional variations (eg the transversal tCho maps) while concentration estimates are in general accord with previous literature. Due to the inclusion of segmentation data in the map calculation, more fringe voxels are filtered out, causing the concentration estimate maps to appear smaller

One dataset (Volunteer 1) was impaired by strong movement artifacts and had to be excluded from further analysis. In five subjects, some ROIs had to be excluded (Table 1), as the GM/WM classification based on *T*_1_‐weighted imaging had failed in these ROIs. In these ROIs, mean concentration estimates were calculated for the remaining subjects. Total Cr SNR and FWHM over all volunteer brain voxels within the quality mask were 11 ± 5 and 0.06 ± 0.02 ppm. Asp and sIns were the only metabolites that were completely excluded from further analysis. Generally, fitting was of good quality, as illustrated by sample spectra in Figure 1 and Supporting Figure 2. Comparison of metabolite maps before *T*_1_ correction and water referencing with concentration estimate maps showed that the concentration estimate maps showed a slight reduction of inhomogeneities and fewer outliers at the brain periphery (Figure 2). The latter was related to the inclusion of GM/WM segmentation, which removed CSF‐dominant voxels from the maps. As our extensive 3D metabolite maps cannot satisfactorily be displayed with a limited number of figures, we supplied multiple complete datasets for review (see Section 4.5).

### 3.2 Quantification quality within ROIs

#### 3.2.1 Regions

Of the 55 segmented regions, 18 fulfill the criteria for “good,” 26 for “acceptable,” and 11 were rejected, as detailed in Table 3, including the mean ROI sizes. Concentration estimate standard deviations are generally higher for smaller regions. The rejected regions were mostly situated in the lower parts of the brain, were small, or were close to the nasal cavities/eyes. Of all metabolites, tCho, tCr, Glu, mIns, and NAA fulfilled the qualification criterion of being fit in more than 66% of voxels (Table 3). An overview over tCr SNR, FWHM, and metabolite CRLBs in relation to the resulting concentration estimate maps is given in Supporting Figure 3.

#### 3.2.2 Metabolites

Of all cortices, the parietal, motor, and cingulate cortices performed the best.

### 3.3 Quantification estimates

**FIGURE 4:**
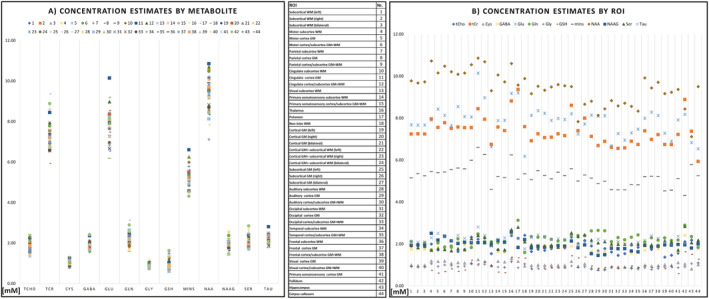
Scatterplots of the metabolite concentration estimates presented in Table 4 and Supporting Table 3. A, Estimated concentrations per metabolite; B, estimated concentrations per ROI

Our concentration estimates for the five qualified metabolites over 44 ROIs are presented in Table 4 and graphically summarized in Figure 4. The highest apparent concentrations were found for tCr, Glu, and NAA. Over all ROIs, the minimum and maximum obtained means [mM] were 1.37‐2.42 for tCho, 5.93‐9.36 for tCr, 6.18‐10.14 for Glu, 4.31‐6.60 for mIns, and 7.12‐10.86 for NAA. We found a high variability between different ROIs. Our estimates were generally within the range of published research for all metabolites. A comparison of our results with previously published concentration estimates^35^, ^37^, ^59^ is shown in Table 5. Metabolite ratios to tCr per ROI are summarized in Supporting Table 1.

**TABLE 4:** Mean concentration estimates per ROI [mM] and their standard deviations for all qualified metabolites in all qualified ROIs

ROI	tCho	tCr	Glu	mIns	NAA
Subcortical WM (left)	1.89 ± 0.76	7.24 ± 2.72	7.68 ± 3.35	5.15 ± 1.92	9.78 ± 3.77
Subcortical WM (right)	1.95 ± 0.71	7.25 ± 2.50	7.67 ± 3.23	5.35 ± 1.77	9.67 ± 3.44
Subcortical WM (bilateral)	1.92 ± 0.73	7.24 ± 2.62	7.67 ± 3.29	5.24 ± 1.85	9.73 ± 3.61
Motor subcortex WM	2.00 ± 0.47	7.96 ± 1.61	7.90 ± 2.25	5.45 ± 1.15	10.72 2.11
Motor cortex GM	1.71 ± 0.50	7.55 ± 2.06	8.42 ± 2.58	5.39 ± 1.72	10.15 ± 2.79
Motor cortex/subcortex GM+WM	1.87 ± 0.50	7.78 ± 1.83	8.13 ± 2.41	5.42 ± 1.43	10.48 ± 2.44
Parietal subcortex WM	1.84 ± 0.51	7.51 ± 2.00	7.64 ± 2.61	5.54 ± 1.38	10.21 ± 2.66
Parietal cortex GM	1.64 ± 0.50	7.57 ± 2.23	8.55 ± 2.81	5.61 ± 1.63	10.09 ± 3.02
Parietal cortex/subcortex GM+WM	1.74 ± 0.52	7.54 ± 2.11	8.07 ± 2.74	5.58 ± 1.50	10.15 ± 2.83
Cingulate subcortex WM	2.32 ± 0.81	7.55 ± 2.75	8.06 ± 3.70	5.99 ± 2.01	10.55 ± 3.57
Cingulate cortex GM	2.22 ± 0.74	8.45 ± 2.62	10.14 ± 3.57	6.60 ± 2.05	10.86 ± 3.41
Cingulate cortex/subcortex GM+WM	2.28 ± 0.78	7.94 ± 2.73	8.98 ± 3.79	6.26 ± 2.05	10.68 ± 3.51
Visual subcortex WM	1.54 ± 0.57	6.74 ± 2.44	6.64 ± 3.18	4.60 ± 1.62	9.31 ± 3.69
Primary somatosensory subcortex WM	1.73 ± 0.50	7.57 ± 1.93	7.88 ± 2.27	5.21 ± 1.27	10.06 ± 2.57
Primary somatosensory cortex/subcortex GM+WM	1.65 ± 0.52	7.40 ± 2.14	7.90 ± 2.48	5.16 ± 1.46	9.73 ± 2.84
Thalamus	2.40 ± 0.83	8.81 ± 3.07	9.18 ± 4.15	6.24 ± 2.21	10.60 ± 4.00
Putamen	2.27 ± 0.86	9.36 ± 3.32	9.20 ± 3.59	5.09 ± 2.04	9.58 ± 3.77
Non‐lobe WM	2.42 ± 0.76	7.59 ± 2.53	6.18 ± 2.75	5.34 ± 1.71	9.88 ± 3.14
Cortical GM (left)	1.64 ± 0.76	6.92 ± 3.06	8.13 ± 3.99	5.06 ± 2.28	9.16 ± 4.47
Cortical GM (right)	1.75 ± 0.64	7.25 ± 2.58	8.35 ± 3.35	5.49 ± 1.91	9.52 ± 3.49
Cortical GM (bilateral)	1.69 ± 0.71	7.07 ± 2.85	8.23 ± 3.70	5.26 ± 2.12	9.33 ± 4.05
Cortical GM+ subcortical WM (left)	1.78 ± 0.77	7.09 ± 2.89	7.89 ± 3.67	5.11 ± 2.10	9.49 ± 4.14
Cortical GM+ subcortical WM (right)	1.86 ± 0.69	7.25 ± 2.54	7.98 ± 3.31	5.41 ± 1.84	9.60 ± 3.47
Cortical GM+ subcortical WM (bilateral)	1.81 ± 0.73	7.16 ± 2.73	7.93 ± 3.50	5.25 ± 1.98	9.54 ± 3.83
Subcortical GM (left)	2.32 ± 0.95	8.61 ± 3.64	8.20 ± 4.06	5.58 ± 2.48	9.50 ± 4.21
Subcortical GM (right)	2.02 ± 0.97	7.40 ± 3.68	7.24 ± 4.16	4.99 ± 2.48	7.83 ± 4.32
Subcortical GM (bilateral)	2.17 ± 0.97	7.99 ± 3.72	7.72 ± 4.15	5.28 ± 2.51	8.66 ± 4.35
Auditory subcortex WM	1.91 ± 0.72	7.13 ± 2.48	8.14 ± 3.23	5.13 ± 1.79	8.81 ± 3.45
Auditory cortex GM	1.67 ± 0.69	6.69 ± 2.60	8.10 ± 3.43	4.88 ± 1.95	8.13 ± 3.56
Auditory cortex/subcortex GM+WM	1.78 ± 0.71	6.89 ± 2.56	8.11 ± 3.34	4.99 ± 1.88	8.44 ± 3.53
Occipital subcortex WM	1.61 ± 0.68	6.61 ± 2.66	6.68 ± 3.35	4.59 ± 1.79	8.83 ± 3.91
Occipital cortex GM	1.46 ± 0.65	6.55 ± 2.92	7.26 ± 3.72	4.57 ± 2.00	8.58 ± 4.31
Occipital cortex/subcortex GM+WM	1.54 ± 0.67	6.58 ± 2.79	6.95 ± 3.55	4.58 ± 1.89	8.71 ± 4.12
Temporal subcortex WM	1.95 ± 0.89	6.89 ± 3.17	7.30 ± 3.68	4.83 ± 2.15	8.56 ± 4.06
Temporal cortex/subcortex GM+WM	1.84 ± 0.87	6.74 ± 3.19	7.47 ± 3.91	4.82 ± 2.21	8.33 ± 4.63
Frontal subcortex WM	1.99 ± 0.79	7.39 ± 2.69	7.99 ± 3.41	5.20 ± 1.92	9.91 ± 3.81
Frontal cortex GM	1.74 ± 0.79	6.98 ± 3.01	8.29 ± 3.98	5.22 ± 2.29	9.44 ± 3.98
Frontal cortex/subcortex GM+WM	1.88 ± 0.80	7.21 ± 2.84	8.12 ± 3.67	5.21 ± 2.09	9.70 ± 3.89
Visual cortex GM	1.37 ± 0.55	6.74 ± 2.72	7.28 ± 3.46	4.53 ± 1.81	9.17 ± 3.99
Visual cortex/subcortex GM+WM	1.46 ± 0.57	6.73 ± 2.57	6.90 ± 3.32	4.56 ± 1.71	9.23 ± 3.83
Primary somatosensory cortex GM	1.57 ± 0.53	7.22 ± 2.33	7.91 ± 2.69	5.11 ± 1.63	9.37 ± 3.06
Pallidum	2.11 ± 0.94	8.88 ± 3.75	8.18 ± 4.30	4.31 ± 2.03	8.43 ± 4.06
Hippocampus	2.18 ± 1.01	7.37 ± 3.35	6.83 ± 3.67	5.78 ± 2.74	7.12 ± 3.73
Corpus callosum	2.07 ± 0.88	5.93 ± 2.62	6.54 ± 3.46	5.25 ± 2.31	9.50 ± 4.28
Mean	1.88	7.37	7.85	5.23	9.43
Min	1.37	5.93	6.18	4.31	7.12
Max	2.42	9.36	10.14	6.60	10.86

**TABLE 5:** Overview of concentration estimation results of this study compared with those in the literature. A breakdown of reference concentration estimate and literature source ROIs is given in Section 4. An analysis per ROI is presented in Table 7

Metabolite	This study: lowest [mM]	Literature: lowest [mM]	This study: highest [mM]	Literature: highest[mM]	Agreement	References
tCho	1.4	0.5	2.4	4	High	Kreis 1993, Hetherington 1994, Pouwels 1998, Gasparovic 2006, Minati 2010, van de Bank 2015, Lecocq 2015, Volk 2018
tCr	5.9	1.8	9.4	14	High	Kreis 1993, Hetherington 1994, Pouwels 1998, Gasparovic 2006, Minati 2010, van de Bank 2015, Lecocq 2015, Volk 2018, Dhamala 2019
GABA	1.6	1.3	2.5	3.5	High	van Zijl 1997, van de Bank 2015, Dhamala 2019, Gonen 2020
Glu	6.2	5	10.1	12	High	Pouwels 1998, Choi 2006, van de Bank 2015, Volk 2018, Dhamala 2019, Gonen 2020
Gln	1.6	1	3.1	5	High	Pouwels 1998, Choi 2006, van de Bank 2015, Dhamala 2019
Gly	0.8	1	1.2	1	High	van Zijl 1997
GSH	0.6	0.7	1.6	2.2	Moderate	Terpstra 2005, Emir 2011, van de Bank 2015, Rai 2018, Dhamala 2019, Gonen 2020
mIns	4.3	3	6.6	9	High	Kreis 1993, Pouwels 1998, Minati 2010, van de Bank 2015, Lecocq 2015, Volk 2018, Dhamala 2019
NAA	7.1	5	10.9	17	High	Kreis 1993, Hetherington 1994, Pouwels 1998, Gasparovic 2006, Minati 2010, van de Bank 2015, Lecocq 2015, Volk 2018, Dhamala 2019
NAAG	1.5	0.5	2.6	3	High	Pouwels 1997, Pouwels 1998, Edden 2007, Dhamala 2019
Ser	1.7	—	2.9	—	—	—
Tau	1.8	1.5	2.8	2.3	Moderate	van Zijl 1997, van de Bank 2015

### 3.4 Inter‐subject coefficients of variation

**FIGURE 3:**
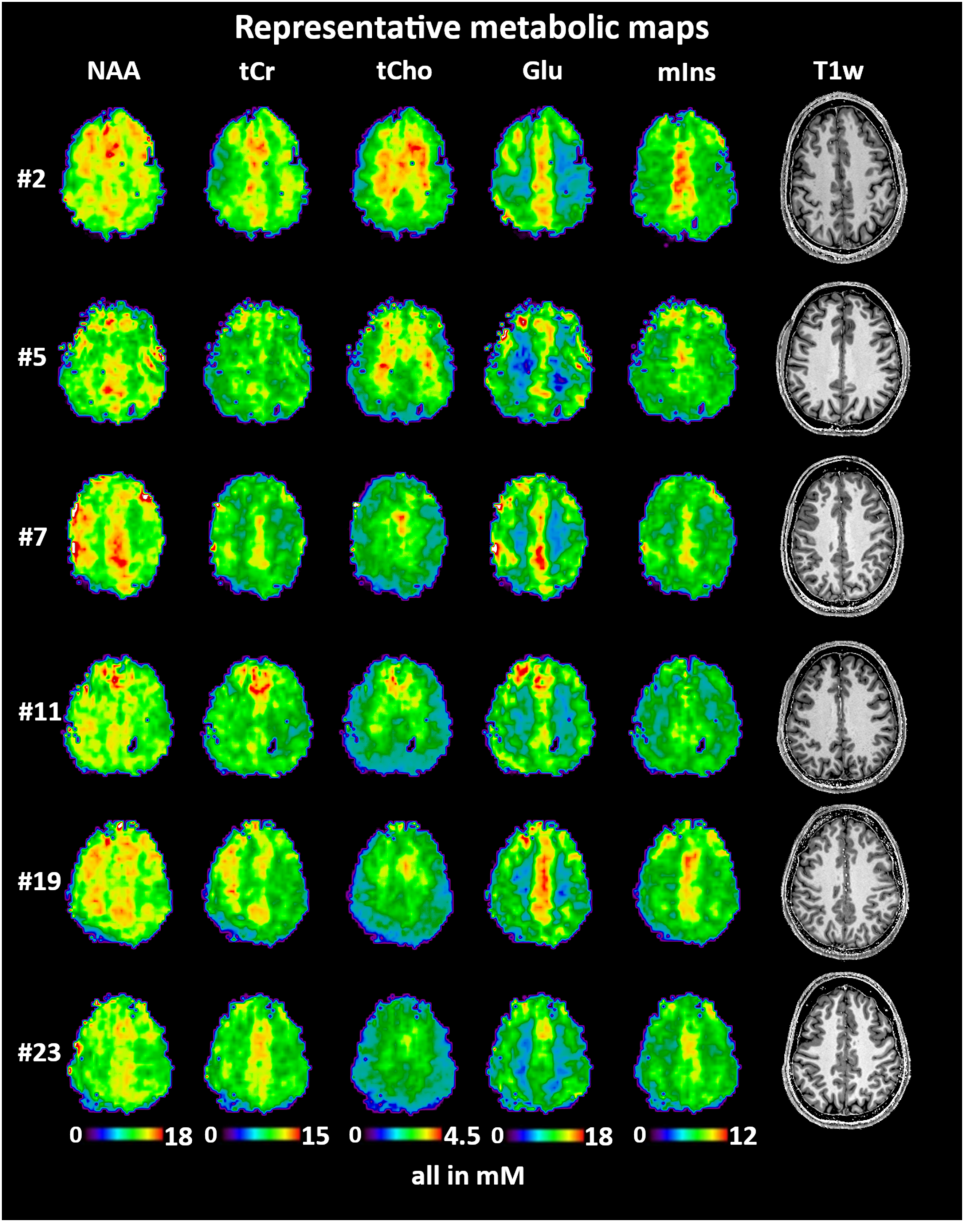
Visualization of data consistency of concentration estimates in six volunteer subjects for NAA, tCr, tCho, Glu, and mIns. Presented is a transversal MRSI slice location directly above the ventricles in all subjects. Full map datasets for a detailed inspection of all metabolite concentration estimate maps of Volunteers 7, 11, 13, 19, and 23 are available at Zenodo (https://doi.org/10.5281/zenodo.5006923)

The inter‐subject CVs in Table 6 show a good comparability of the regional analyses between subjects for most cases. These CVs were the lowest for tCr and NAA and, generally, in the range of 10‐20%. In the majority of “good” ROIs, tCho, tCr, Glu, mIns, and NAA CVs were 10% or less. Figure 3 illustrates these findings over multiple subjects. The CVs for ratios to tCr (Supporting Table 2) were very similar, with differences between the means over all ROIs not exceeding 4%.

**TABLE 6:** Inter‐subject CVs of the concentration estimates per ROI displayed in Table 4. As expected, higher SNR/concentration metabolites corresponded to the lowest CVs

ROI	tCho [%]	tCr [%]	Glu [%]	mIns [%]	NAA [%]
Subcortical WM (left)	7	7	7	8	7
Subcortical WM (right)	6	6	8	6	6
Subcortical WM (bilateral)	6	6	7	7	6
Motor subcortex WM	8	7	7	7	7
Motor cortex GM	6	8	8	8	9
Motor cortex/subcortex GM + WM	7	7	7	8	8
Parietal subcortex WM	11	10	10	10	10
Parietal cortex GM	10	10	11	10	11
Parietal cortex/subcortex GM + WM	10	10	11	10	11
Cingulate subcortex WM	9	10	12	10	9
Cingulate cortex GM	9	10	12	10	10
Cingulate cortex/subcortex GM + WM	9	10	12	10	9
Visual subcortex WM	13	15	14	14	8
Primary somatosensory subcortex WM	8	9	8	10	8
Primary somatosensory cortex/subcortex GM + WM	8	9	8	10	9
Thalamus	19	20	21	19	16
Putamen	9	9	15	13	11
Non‐lobe WM	10	7	15	10	8
Cortical GM (left)	7	8	7	8	8
Cortical GM (right)	6	6	7	6	6
Cortical GM (bilateral)	6	6	7	7	7
Cortical GM + subcortical WM (left)	7	7	7	8	8
Cortical GM + subcortical WM (right)	6	6	7	6	6
Cortical GM + subcortical WM (bilateral)	6	6	7	7	6
Subcortical GM (left)	10	9	14	13	10
Subcortical GM (right)	15	15	16	14	15
Subcortical GM (bilateral)	12	11	15	13	11
Auditory subcortex WM	8	9	9	10	9
Auditory cortex GM	7	8	8	8	9
Auditory cortex/subcortex GM + WM	7	8	9	9	9
Occipital subcortex WM	12	12	12	12	7
Occipital cortex GM	11	12	10	12	7
Occipital cortex/subcortex GM + WM	11	12	11	12	6
Temporal subcortex WM	6	8	7	9	8
Temporal cortex/subcortex GM + WM	5	8	7	8	8
Frontal subcortex WM	8	7	10	9	8
Frontal cortex GM	8	7	9	8	9
Frontal cortex/subcortex GM + WM	8	7	9	9	8
Visual cortex GM	13	17	12	15	8
Visual cortex/subcortex GM + WM	13	16	12	14	7
Primary somatosensory cortex GM	8	10	9	10	9
Pallidum	14	12	24	15	19
Hippocampus	16	17	18	18	16
Corpus callosum	16	16	19	15	17
Mean	9	10	11	10	9
Min.	5	6	7	6	6
Max.	19	20	24	19	19

## 4 DISCUSSION

We have assessed the average and region‐specific concentration estimates, as well as their inter‐subject variability, for five neuro‐metabolites that can be reliably mapped in the human brain using our 3D‐CRT‐based FID‐MRSI sequence at 7 T. To improve upon previous work,^21^ we have expanded the subject cohort, also evaluated less abundant metabolites, and assessed concentration estimates instead of ratios and data quality over a large number of automatically segmented brain ROIs to provide a more detailed understanding of the performance of 7 T 3D‐CRT‐based FID‐MRSI. The additional inclusion of *T*_1_ corrections and internal water referencing allowed a more quantitative assessment (ie of concentration estimates). In summary, we found our method to yield acceptable results in 23 of 24 volunteer subjects for five metabolites in 44 predefined ROIs. Our concentration estimates are in the range of previous reports, while inter‐subject CVs indicated a good level of stability in many of these metabolites and ROIs. Still, the quantification quality of GABA, Gln, Gly, GSH, NAAG, Ser, and Tau in healthy subjects cannot be considered sufficient for this MRSI application, necessitating different approaches or methodological improvements if these are required. These results are important to define the limits of stability, sensitivity, and regional reliability for our MRSI method for future research applications. As the sum of our generated imaging data is hard to convey within few figures, we invite the readers to look at the supplementary full datasets provided by us on Zenodo.

To contextualize our resultant metabolite distributions, we conducted an extensive comparison with previous research. Concentration estimates for many metabolites in many brain regions have been reported over the last decades, sometimes with contradicting results due to different processing methods, subject cohorts, partial volume effects, acquisition schemes (eg MRSI or single‐voxel spectroscopy, SVS), and scanner types. Different quantification algorithms are known to affect reported results.^60^ Dhamala et al^37^ found that correlations varied strongly for different metabolites between different MRS methods in the same subjects. A comparison with our 7 T‐FID‐MRSI method with 3.4 mm nominal isometric resolution, a *T*_R_ of 450 ms, and an acquisition delay of 1.3 ms remains challenging. Nonetheless, our results are, overall, consistent with previously reported concentration estimates^31^, ^32^, ^33^, ^34^, ^35^, ^36^, ^37^, ^53^, ^61^, ^62^, ^63^, ^64^, ^65^, ^66^, ^67^, ^68^, ^69^ as summarized in Table 5, and mostly found in the middle or lower range of references. For metabolites separable in our study, such as Glu/Gln and NAA/NAAG, they individually agree with previous literature (Tables 5 and7), and further comparing their sums agrees well with studies that cannot separate them.

**TABLE 7:** Comparison of this study's concentration estimates and their standard deviations in specific ROIs to literature. The details of this comparison are elaborated on in Section 4

Source	tCho	tCr	Glu	mIns	NAA
*Occipital GM*					
This study	1.46 ± 0.65	6.55 ± 2.92	7.26 ± 3.72	4.57 ± 2.00	8.58 ± 4.31
Kreis 1993	1.41 ± 0.05	7.95 ± 0.11	—	6.24 ± 0.21	9.06 ± 0.12
Pouwels 1998	0.88 ± 0.10	6.90 ± 0.70	8.60 ± 1.10	4.10 ± 0.60	9.20 ± 0.90
Lecocq 2015	1.20‐1.40	7.30 8.70	—	3.50‐4.60	9.50‐12.10
*Occipital WM*					
This study	1.61 ± 0.68	6.61 ± 2.66	6.68 ± 3.35	4.59 ± 1.79	8.83 ± 3.91
Pouwels 1998	1.64 ± 0.21	5.50 0.80	6.00 ± 1.20	4.10 ± 0.80	7.80 ± 0.90
Lecocq 2015	1.20‐1.40	7.30 8.70	—	3.50‐4.60	9.50‐12.10
*Frontal GM*					
This study	1.74 ± 0.79	6.98 ± 3.01	8.29 ± 3.98	5.22 ± 2.29	9.44 ± 3.98
Pouwels 1998	1.38 ± 0.17	6.40 ± 0.70	8.50 ± 1.00	4.30 ± 0.90	7.70 ± 1.00
Lecocq 2015	1.50 ± 2.50	6.70 ± 8.60	—	4.00 ± 7.80	9.50 ± 12.50
*Frontal WM*					
This study	1.99 ± 0.79	7.39 ± 2.69	7.99 ± 3.41	5.20 ± 1.92	9.91 ± 3.81
Pouwels 1998	1.78 ± 0.41	5.70 ± 0.50	7.00 ± 2.60	3.80 ± 0.90	8.10 ± 0.90
Minati 2010	3.60 ± 0.80	11.50 ± 2.40	—	7.60 ± 2.00	14.20 ± 2.00
Lecocq 2015	1.50 ± 2.50	6.70 ± 8.60	—	4.00 ± 7.80	9.50 ± 12.50
*Parietal lobe*					
This study	1.74 ± 0.52	7.54 ± 2.11	8.07 ± 2.74	5.58 ± 1.50	10.15 ± 2.83
Lecocq 2015	0.80‐1.10	4.10‐6.50	—	1.90‐3.20	8.30‐10.70
Volk 2018	1.65 ± 0.05	7.54 ± 0.14	12.55 ± 0.22	3.70 ± 0.08	11.59 ± 0.13
*Thalamus*					
This study	2.40 ± 0.83	8.81 ± 3.07	9.18 ± 4.15	6.24 ± 2.21	10.60 ± 4.00
Minati 2010	3.40 ± 0.80	12.00 ± 1.10	—	6.60 ± 1.80	16.30 ± 2.00
Lecocq 2015	1.20‐1.30	5.90‐6.60	—	3.00‐3.30	5.90‐6.60
*Temporal cortex*					
This study	1.84 ± 0.87	6.74 ± 3.19	7.47 ± 3.91	4.82 ± 2.21	8.33 ± 4.63
Minati 2010	3.60 ± 1.10	12.00 ± 4.00	—	7.90 ± 3.00	14.10 ± 2.50
Lecocq 2015	1.40‐2.30	7.50‐8.80	—	4.20‐5.30	8.80‐10.90
*Cingulate subcortex*					
This study	2.28 ± 0.78	7.94 ± 2.73	8.98 ± 3.79	6.26 ± 2.05	10.68 ± 3.51
Hetherington 1994	2.30 ± 0.40	7.70 ± 0.90	—	—	13.50 ± 0.90
van de Bank 2015	1.30 ± 0.10	8.10 ± 0.50	9.40 ± 0.80	6.40 ± 0.60	12.10 ± 1.00
Lecocq 2015	1.00‐2.50	5.90‐9.30	—	3.20‐6.40	7.90‐11.50
Gonen 2020	—	—	10.20 ± 1.80	—	—

Some publications used more ROIs without GM/WM separation per region and the results are therefore presented as a range of their findings.

Going from overall reported values to specific ROIs, as compared in Table 7, we found similar concentrations (within the respective standard deviations of each other) in occipital GM and WM,^31^, ^32^, ^35^ frontal WM,^32^, ^35^ parietal lobe,^36^ thalamus,^34^ temporal cortex,^35^ and cingulate cortex^33^, ^63^ for most studies. We found disagreements beyond a standard deviation for the cingulate cortex with van de Bank et al,^68^ for the frontal WM, thalamus, and temporal cortex with Minati et al,^34^ and for the parietal cortex as well as the thalamus with Lecocq et al.^35^

For metabolite ratios to tCr, we also compared our results with the 7 T MRSI results of Bhogal et al,^70^ as seen in Table 8. Over the six compared ROIs, our ratios are consistently higher for all except GSH/tCr, which is mixed. The effect is most pronounced for Ins + Gly. The generally higher ratios could be sourced in lower quantification estimates of tCr or differences in the MRSI acquisition.

**TABLE 8:** Comparison of this study's metabolite ratios and standard deviations of tCho, Glu, Glu + Gln, NAA + NAAG, Ins + Gly, and GSH to tCr in six ROIs compared with similar 7 T MRSI results of Reference.^70^ The results of our study are consistently higher for all except GSH/tCr, with two higher/lower/same ratio regions each

Source	tCho/tCr	Glu/tCr	(Glu + Gln)/tCr	(NAA + NAAG)/tCr	(Ins + Gly)/tCr	GSH/tCr
*(Cortical) GM*						
This study	0.24 ± 0.01	1.17 ± 0.09	1.50 ± 0.10	1.59 ± 0.09	0.86 ± 0.04	0.11 ± 0.01
Bhogal 2020	0.17 ± 0.05	0.97 ± 0.20	1.20 ± 0.27	1.13 ± 0.28	0.41 ± 0.16	0.15 ± 0.06
*(Subcortical) WM*						
This study	0.27 ± 0.02	1.07 ± 0.09	1.34 ± 0.10	1.62 ± 0.09	0.85 ± 0.04	0.12 ± 0.01
Bhogal 2020	0.22 ± 0.04	0.72 ± 0.14	0.88 ± 0.17	1.27 ± 0.20	0.41 ± 0.11	0.15 ± 0.04
*Corpus callosum*						
This study	0.35 ± 0.03	1.13 ± 0.28	1.38 ± 0.10	1.97 ± 0.27	1.06 ± 0.13	0.21 ± 0.04
Bhogal 2020	0.23 ± 0.04	0.73 ± 0.13	0.90 ± 0.16	1.26 ± 0.22	0.45 ± 0.09	0.17 ± 0.04
*Pallidum*						
This study	0.24 ± 0.02	0.90 ± 0.17	1.20 ± 0.20	1.15 ± 0.15	0.56 ± 0.07	0.17 ± 0.03
Bhogal 2020	0.18 ± 0.01	0.58 ± 0.10	0.71 ± 0.12	0.85 ± 0.19	0.31 ± 0.06	0.15 ± 0.04
*Thalamus*						
This study	0.28 ± 0.03	1.07 ± 0.24	1.34 ± 0.26	1.53 ± 0.16	0.83 ± 0.14	0.15 ± 0.04
Bhogal 2020	0.22 ± 0.03	0.75 ± 0.10	0.93 ± 0.12	1.07 ± 0.12	0.36 ± 0.09	0.15 ± 0.02
*Putamen*						
This study	0.24 ± 0.01	0.98 ± 0.12	1.30 ± 0.14	1.23 ± 0.08	0.63 ± 0.05	0.14 ± 0.03
Bhogal 2020	0.19 ± 0.02	0.78 ± 0.10	0.97 ± 0.13	0.84 ± 0.14	0.32 ± 0.06	0.15 ± 0.03

For some metabolites, previous concentration estimates are only reported sparsely. Our 0.76‐1.16 mM of Gly align well to the 1.02 mM in Reference^64^ and to a Gly/tCr of 0.14 in Reference,^71^ which compares well with our range of 0.08‐0.17 for Gly/tCr. We established concentrations of 1.68‐2.85 mM for Ser, with a ratio to tCr of 0.8 in Reference,^72^ which that was published in the MRS literature. Our Ser/tCr of 0.21‐0.36 is notably lower. For Tau, we found 1.77‐2.81 mM, which is higher than the 1.48 mM reported in Reference,^64^ but well aligned with the 2.3 mM for Tau + glucose in Reference.^68^

Considering the multitude of applied methods and overall limited sample sizes, our results are in general agreement with the current state of knowledge in the field but are for the first time based on concentration estimation for high‐resolution 7 T FID‐MRSI. We see a further need to investigate metabolites such as GSH, Ser, and Tau, which are difficult to quantify and for which MRS‐based concentration estimates are scarce.

Our inter‐subject CVs were the smallest for metabolites with the highest SNR (ie, NAA, tCr, tCho, Glu, and mIns are in the <10% range) but approached 30% for other metabolites in some ROIs (see Table 6). Comparison with the literature is difficult, as most studies report intra‐subject or inter‐system CVs, such as van de Bank et al,^68^ who reported CVs for SVS on four different 7 T systems in the posterior cingulate cortex of 3‐4% for NAA, tCho, tCr, mIns, and Glu, 22.2% for GABA, 14.4% for GSH, and 8.8% for Gln. Another example, but at 3 T, is the study by Zhang et al,^73^ who calculated intra‐subject CVs for whole‐brain EPSI in 10 volunteers over three scans in 47 ROIs and found NAA CVs of 3.3%‐17.8%, tCho CVs of 3.7%‐31.0%, tCr CVs of 3.1%‐18.0% (with mean ROI CVs < 10%), and mIns CVs of 5.9%‐54.0%. Inter‐subject CVs for metabolic ratios at 3 T were reported by Veenith et al,^74^ with mean CVs of 21.24% for tCho/tCr and 13.30% for tNAA/tCr. Except for higher mIns CVs, these results are similar to our findings, but our 7 T MRSI featured a higher resolution and more quantifiable metabolites and was also affected by additional physiologic inter‐subject variation. Considering that the CV calculation did not account for diurnal effects,^36^ or age^75^, ^76^, ^77^ and sex differences,^78^, ^79^ these results seem convincing but still include methodological artifacts such as subject motion. Intra‐subject CVs from a test‐retest study will be necessary to complete the picture. In another MRSI study at 3 T, we found that the application of motion correction improved the CVs of metabolite ratios to tCr by 30%.^30^ Another source of local variability would be the combination of *T*_1_ weighting with our short *T*_R_ and a lack of knowledge of local tissue metabolite *T*_1_ values.^52^, ^80^ Although we tried to correct our *T*_1_ estimates and reference water concentrations for voxel‐wise GM/WM fractions, we assume that additional variation exists based on this.^53^ More precise concentration estimates could be obtained via the direct mapping of tissue water content.^81^ Due to our echo‐less acquisition approach with negligible acquisition delay, our results can be considered to be robust to *T*_2_ effects. This is a potential advantage in the study of the aging brain or of pathologies that can cause local iron deposits, which affect metabolite and water *T*_2_ values.^82^

### 4.1 Limitations

Our study has some limitations. *T*_1_ values for multiple metabolites had to be estimated without previous reports. The assumption of a single metabolite *T*_1_ over the whole brain is also a rough approximation. This could have biased the estimated concentrations.

Quantification is further limited by the precision of the internal water referencing, which relies on the assumption of tissue water relaxation times and concentrations, effective local excitation, and water signal quantification. Beyond the general variability of MRS quantification results based on the fit model parameters, L2 regularization is also known to influence metabolite signal estimation, especially NAA,^83^ even if just by removing lipid signals. The comparison of quantification estimates between different field strengths, acquisition schemes, processing pipelines, resolutions, and brain segmentations limits the comparability to the even subset of MRS studies that report concentration estimates.

Our study is limited to the analysis of inter‐subject variations and does not, therefore, report on intra‐subject variations. Measuring intra‐subject variation could also help to separate the more subject‐specific (eg physiological) from the method‐specific contributions to variability.

The exclusion of 11 ROIs, predominantly basal brain regions such as brain stem or cerebellum, from further analysis was necessary due to the lack of spectral fit confidence, limiting the mappable brain coverage. This shows that *B*_0_‐ and *B*_1_‐field inhomogeneities (the first caused by the proximity to the nasal and auditory cavities, the second by the limitations of single channel transmit coils at 7 T) remain a significant ultra‐high‐field challenge, which will have to be resolved via hardware improvements^84^, ^85^, ^86^ and/or further improved high‐resolution approaches.^87^

Filtering of outliers based on a set of rigid criteria is insufficient. In the future, automated quality assessment of voxels based on deep learning will be necessary to evaluate datasets of this scale adequately. The necessary interpolation between MRSI and reference imaging combined with MRSI partial volume effects, even at our high resolution, are further confounding factors for the regional analysis as relevant GM/WM fractions remain within the ROIs (Table 3), reducing the expected GM/WM contrast, eg for Glu. While we obtained results for difficult‐to quantify metabolites in healthy tissue (ie GABA, Gln, Gly, GSH, NAAG, Ser, and Tau), the percentage of voxels within the quality criteria remained overall low (Table 1).

Subject motion is another factor not yet accounted for in our study, and significant improvements are expected in stability,^88^, ^89^ which is of particular importance for studies in children and elderly patients.^26^ In particular, real‐time correction has been shown to significantly enhance data quality in high‐resolution 3D‐CRT‐based FID‐MRSI at 3 T.^30^

### 4.2 Conclusions/outlook

We have stablished the brain region‐specific concentration estimates and their variability in a large number of healthy young volunteers for our whole‐brain MRSI‐based metabolic maps at 7 T for the first time. While not all brain ROIs performed well enough to be considered, especially basal regions such as the cerebellum, we could successfully quantify five metabolites—tCho, tCr, NAA, Glu, and Ins—in 44 ROIs, with all others not being quantified with a sufficient quality in a substantial number of voxels. Our estimated concentrations are consistent with previous research.

This was a necessary first step to define the reliability and to guide future basic and clinical research of the brain metabolism and to show the capability of our high‐resolution 3D‐MRSI technique in the current discussion of MRSI standardization.^52^, ^90^, ^91^ Our results can guide future study planning, targeting specific brain regions and metabolites of interest on the one hand and the eventual development of a 7 T‐MRSI based metabolic brain atlas on the other. The next avenues of research could be the investigation of intra‐subject variability, improved quantification by direct water concentration mapping,^81^ also in pathologies, and better definition of specific relaxation times. With these improvements, the metabolites that currently lack reliability could be reevaluated and an in‐depth study of sex and age differences could be carried out. In summary, we have shown new insights into the expectable results and stability of our fast high‐resolution MRSI at 7 T, but this approach still requires more sophistication.

## CONFLICT OF INTEREST

We have no conflicts of interest to report.

## SUPPLEMENTARY MATERIALS/DATA AVAILABILITY STATEMENT

Individual subject concentration estimate map datasets in MINC and NIFTI format including SNR, FWHM, and CRLB maps are available on Zenodo: https://doi.org/10.5281/zenodo.5006923.^92^ A summary of the MRSI method used according to the MRSinMRS experts' consensus^93^ can be found in Supporting Table 5.

## Supporting information


**Figure S1.** Flow chart of the post‐processing and evaluation pipeline. Details for every step can be found in the Experimental section.
**Figure S2**. More sample spectra from different volunteers and brain regions, of different qualities, including a spectrum of the excluded volunteer featuring a dominant lipid artefact at the edge of the excluded fitting range.
**Figure S3.** Examples of MRSI fitting quality markers (tCr SNR and FWHM, metabolite CRLBs) in two volunteers. A) complements data shown in Figure 2. As the CRLB maps show the difficulty of expressing the dynamic range of values, we have also made CRLB maps available for the subject in the supplementary data available at Zenodo.
**Table S1.** Mean ratios to tCr and their standard deviations for all other metabolites, based on the overall ROI concentration estimates.
**Table S2.** Inter‐subject CVs for ratios to tCr per ROI, with high similarity to concentration estimate CVs, as described in Tbl.5.
**Table S3.** Mean concentration estimates per ROI [mM] and their standard deviations for all quantified metabolites in all qualified ROIs.
**Table S4.** Inter‐subject CVs of the concentration estimates per ROI displayed in Sup.Tbl. 3. As expected, higher SNR/concentration metabolites corresponded to the lowest CVs.
**Table S5.** A summary of the MRSI method according to the MRSinMRS expert's consensus proposed standard^93^.

## Data Availability

Individual subject ROI quantification tables, as well as metabolite and concentration estimate map datasets in MINC and NIFTI format, are available at Zenodo https://doi.org/10.5281/zenodo.500692392. A summary of the MRSI method used according to the MRSinMRS expert’s consensus can be found in Sup.Tbl. 5.
