# Supplementary material for: Inter‐subject stability and regional concentration estimates of 3D‐FID‐MRSI in the human brain at 7 T
Source: NMR Biomed. 2021 Aug 11;34(12):e4596. doi: 10.1002/nbm.4596 (PMC11475238; doi:10.1002/nbm.4596)
Supplement: Supplementary file 1 — Figure S1. Flow chart of the post‐processing and evaluation pipeline. Details for every step can be found in the Experimental section. Figure S2. More sample spectra from different volunteers and brain regions, of different qualities, including a spectrum of the excluded volunteer featuring a dominant lipid artefact at the edge of the excluded fitting range. Figure S3. Examples of MRSI fitting quality markers (tCr SNR and FWHM, metabolite CRLBs) in two volunteers. A) complements data shown in Figure 2. As the CRLB maps show the difficulty of expressing the dynamic range of values, we have also made CRLB maps available for the subject in the supplementary data available at Zenodo. Table S1. Mean ratios to tCr and their standard deviations for all other metabolites, based on the overall ROI concentration estimates. Table S2. Inter‐subject CVs for ratios to tCr per ROI, with high similarity to concentration estimate CVs, as described in Tbl.5. Table S3. Mean concentration estimates per ROI [mM] and their standard deviations for all quantified metabolites in all qualified ROIs. Table S4. Inter‐subject CVs of the concentration estimates per ROI displayed in Sup.Tbl. 3. As expected, higher SNR/concentration metabolites corresponded to the lowest CVs. Table S5. A summary of the MRSI method according to the MRSinMRS expert's consensus proposed standard93. [file NBM-34-e4596-s001.docx]

# Supplementary Data


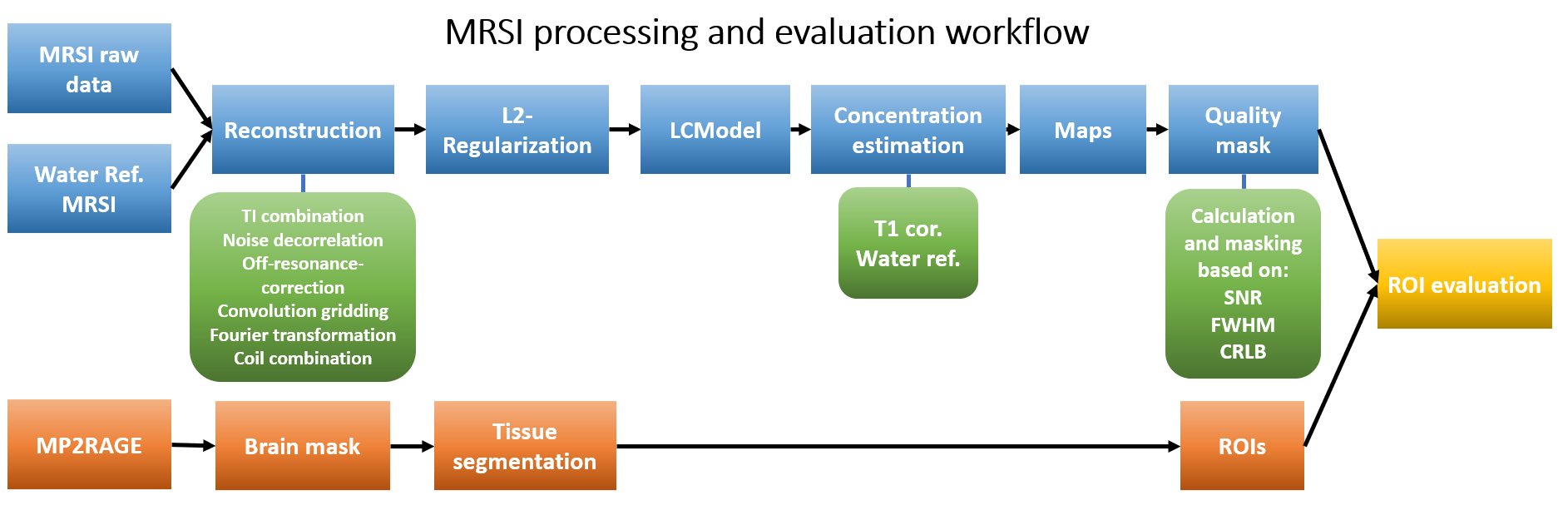


Supplementary Figure 1: Flow chart of the post-processing and evaluation pipeline. Details for every step can be found in the Experimental section.


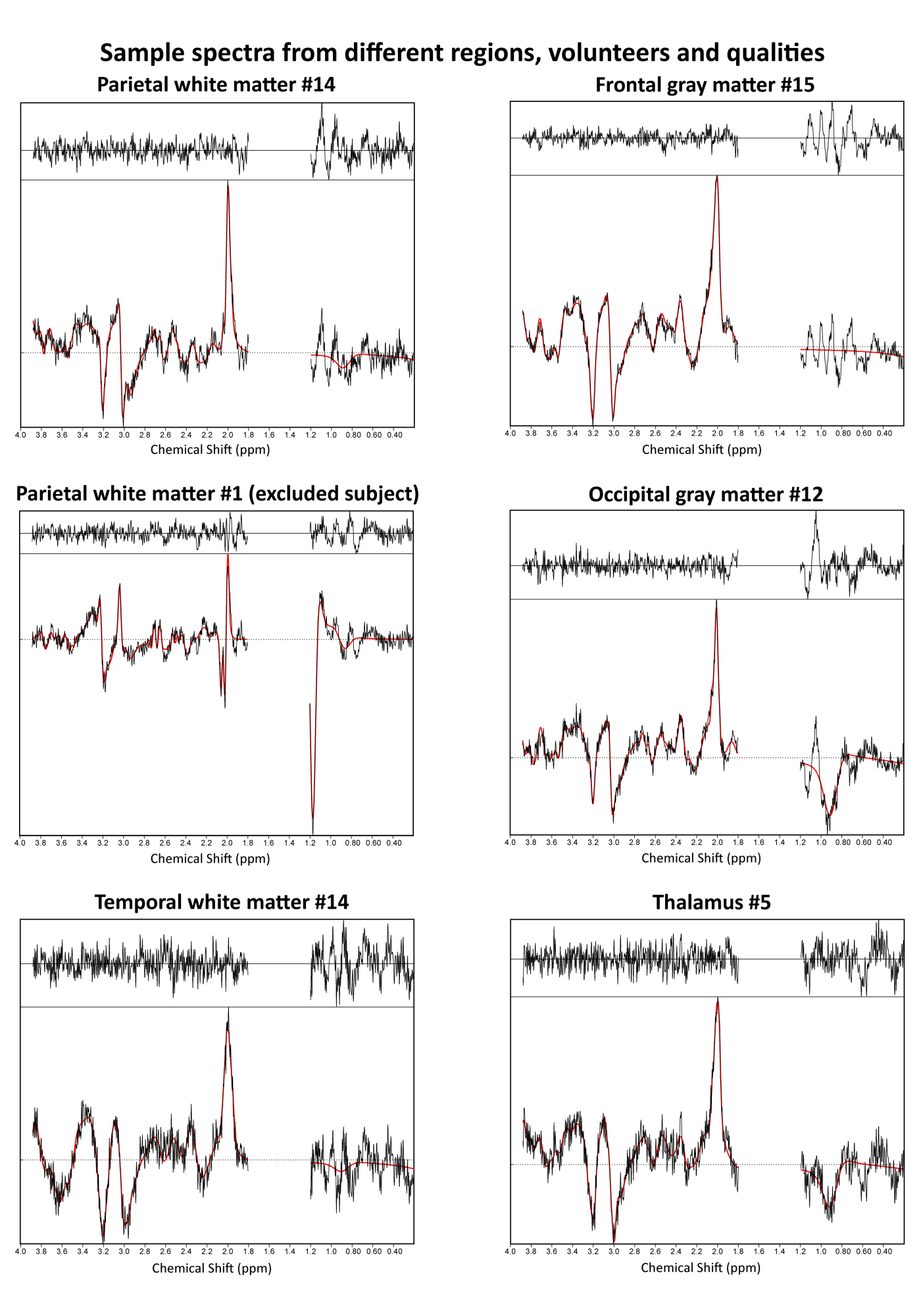


Supplementary Figure 2: More sample spectra from different volunteers and brain regions, of different qualities, including a spectrum of the excluded volunteer featuring a dominant lipid artefact at the edge of the excluded fitting range.


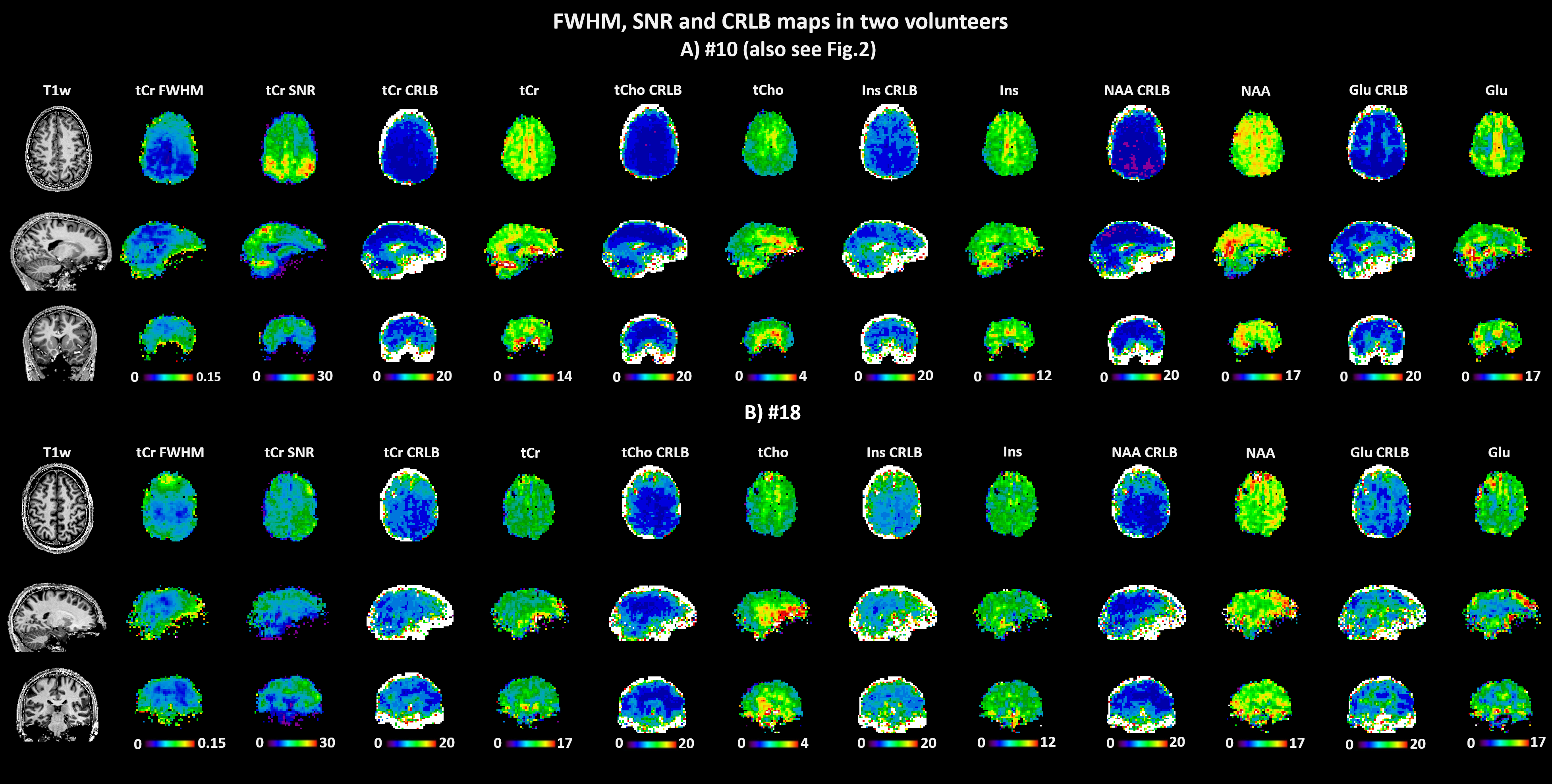
Supplementary Figure 3: Examples of MRSI fitting quality markers (tCr SNR and FWHM, metabolite CRLBs) in two volunteers. A) complements data shown in Fig.2. As the CRLB maps show the difficulty of expressing the dynamic range of values, we have also made CRLB maps available for the subject in the supplementary data available at Zenodo.

| **Supplementary Table 1 - Mean ratios to tCr of allmetabolites for overall ROI values** | | | | | | | | | | | | | | | | | | | | | | | | | | | | | | | | | |
| --- | --- | --- | --- | --- | --- | --- | --- | --- | --- | --- | --- | --- | --- | --- | --- | --- | --- | --- | --- | --- | --- | --- | --- | --- | --- | --- | --- | --- | --- | --- | --- | --- | --- |
| **ROI** | **tCho** | | | **GABA** | | | **Glu** | | | **Gln** | | | **Gly** | | | **GSH** | | | **mIns** | | | **NAA** | | | **NAAG** | | | **Ser** | | | **Tau** | | |
| **Subcortical WM (left)** | 0.26 | ± | 0.02 | 0.24 | ± | 0.04 | 1.07 | ± | 0.08 | 0.28 | ± | 0.06 | 0.12 | ± | 0.02 | 0.13 | ± | 0.01 | 0.71 | ± | 0.04 | 1.36 | ± | 0.09 | 0.27 | ± | 0.03 | 0.27 | ± | 0.04 | 0.29 | ± | 0.06 |
| **Subcortical WM (right)** | 0.27 | ± | 0.02 | 0.24 | ± | 0.04 | 1.07 | ± | 0.10 | 0.27 | ± | 0.05 | 0.12 | ± | 0.01 | 0.12 | ± | 0.01 | 0.74 | ± | 0.05 | 1.34 | ± | 0.09 | 0.27 | ± | 0.03 | 0.25 | ± | 0.04 | 0.29 | ± | 0.04 |
| **Subcortical WM (bilateral)** | 0.27 | ± | 0.02 | 0.24 | ± | 0.04 | 1.07 | ± | 0.09 | 0.27 | ± | 0.05 | 0.12 | ± | 0.01 | 0.12 | ± | 0.01 | 0.73 | ± | 0.04 | 1.35 | ± | 0.09 | 0.27 | ± | 0.03 | 0.26 | ± | 0.03 | 0.29 | ± | 0.05 |
| **Motor subcortex WM** | 0.25 | ± | 0.02 | 0.22 | ± | 0.04 | 1.00 | ± | 0.09 | 0.19 | ± | 0.06 | 0.13 | ± | 0.01 | 0.09 | ± | 0.01 | 0.69 | ± | 0.04 | 1.36 | ± | 0.10 | 0.31 | ± | 0.03 | 0.21 | ± | 0.04 | 0.29 | ± | 0.04 |
| **Motor cortex GM** | 0.23 | ± | 0.01 | 0.24 | ± | 0.04 | 1.12 | ± | 0.09 | 0.26 | ± | 0.07 | 0.12 | ± | 0.01 | 0.08 | ± | 0.01 | 0.71 | ± | 0.04 | 1.35 | ± | 0.10 | 0.29 | ± | 0.03 | 0.24 | ± | 0.04 | 0.33 | ± | 0.04 |
| **Motor cortex/subcortex GM+WM** | 0.24 | ± | 0.02 | 0.23 | ± | 0.04 | 1.05 | ± | 0.09 | 0.22 | ± | 0.06 | 0.13 | ± | 0.01 | 0.09 | ± | 0.01 | 0.70 | ± | 0.04 | 1.36 | ± | 0.10 | 0.30 | ± | 0.03 | 0.22 | ± | 0.04 | 0.30 | ± | 0.04 |
| **Parietal subcortex WM** | 0.24 | ± | 0.01 | 0.25 | ± | 0.07 | 1.02 | ± | 0.08 | 0.24 | ± | 0.05 | 0.13 | ± | 0.02 | 0.10 | ± | 0.01 | 0.74 | ± | 0.04 | 1.36 | ± | 0.08 | 0.29 | ± | 0.03 | 0.22 | ± | 0.04 | 0.28 | ± | 0.04 |
| **Parietal cortex GM** | 0.22 | ± | 0.01 | 0.27 | ± | 0.07 | 1.13 | ± | 0.08 | 0.29 | ± | 0.06 | 0.12 | ± | 0.02 | 0.09 | ± | 0.01 | 0.74 | ± | 0.04 | 1.34 | ± | 0.08 | 0.27 | ± | 0.03 | 0.23 | ± | 0.04 | 0.32 | ± | 0.04 |
| **Parietal cortex/subcortex GM+WM** | 0.23 | ± | 0.01 | 0.26 | ± | 0.07 | 1.08 | ± | 0.08 | 0.26 | ± | 0.06 | 0.12 | ± | 0.02 | 0.10 | ± | 0.01 | 0.74 | ± | 0.04 | 1.35 | ± | 0.08 | 0.28 | ± | 0.03 | 0.22 | ± | 0.04 | 0.30 | ± | 0.04 |
| **Cingulate subcortex WM** | 0.31 | ± | 0.02 | 0.25 | ± | 0.07 | 1.08 | ± | 0.16 | 0.23 | ± | 0.06 | 0.14 | ± | 0.02 | 0.16 | ± | 0.03 | 0.80 | ± | 0.10 | 1.41 | ± | 0.12 | 0.27 | ± | 0.03 | 0.30 | ± | 0.04 | 0.27 | ± | 0.06 |
| **Cingulate cortex GM** | 0.27 | ± | 0.02 | 0.28 | ± | 0.06 | 1.21 | ± | 0.14 | 0.29 | ± | 0.06 | 0.12 | ± | 0.01 | 0.12 | ± | 0.02 | 0.79 | ± | 0.08 | 1.29 | ± | 0.08 | 0.24 | ± | 0.03 | 0.28 | ± | 0.04 | 0.33 | ± | 0.05 |
| **Cingulate cortex/subcortex GM+WM** | 0.29 | ± | 0.02 | 0.26 | ± | 0.06 | 1.14 | ± | 0.15 | 0.26 | ± | 0.06 | 0.13 | ± | 0.02 | 0.14 | ± | 0.02 | 0.79 | ± | 0.09 | 1.36 | ± | 0.10 | 0.26 | ± | 0.03 | 0.29 | ± | 0.04 | 0.30 | ± | 0.05 |
| **Visual subcortex WM** | 0.23 | ± | 0.03 | 0.29 | ± | 0.12 | 1.02 | ± | 0.17 | 0.30 | ± | 0.08 | 0.14 | ± | 0.03 | 0.14 | ± | 0.03 | 0.69 | ± | 0.06 | 1.43 | ± | 0.20 | 0.28 | ± | 0.08 | 0.32 | ± | 0.07 | 0.29 | ± | 0.07 |
| **Primary somatosensory subcortex WM** | 0.23 | ± | 0.01 | 0.22 | ± | 0.05 | 1.05 | ± | 0.09 | 0.25 | ± | 0.07 | 0.12 | ± | 0.02 | 0.08 | ± | 0.01 | 0.69 | ± | 0.05 | 1.34 | ± | 0.11 | 0.29 | ± | 0.04 | 0.23 | ± | 0.04 | 0.29 | ± | 0.05 |
| **Primary somatosensory cortex/subcortex GM+WM** | 0.22 | ± | 0.01 | 0.22 | ± | 0.04 | 1.08 | ± | 0.10 | 0.27 | ± | 0.07 | 0.12 | ± | 0.02 | 0.08 | ± | 0.01 | 0.70 | ± | 0.05 | 1.33 | ± | 0.11 | 0.28 | ± | 0.04 | 0.23 | ± | 0.03 | 0.31 | ± | 0.05 |
| **Thalamus** | 0.28 | ± | 0.03 | 0.27 | ± | 0.09 | 1.07 | ± | 0.24 | 0.27 | ± | 0.10 | 0.12 | ± | 0.03 | 0.15 | ± | 0.04 | 0.71 | ± | 0.13 | 1.24 | ± | 0.15 | 0.29 | ± | 0.07 | 0.31 | ± | 0.08 | 0.25 | ± | 0.10 |
| **Putamen** | 0.24 | ± | 0.01 | 0.19 | ± | 0.05 | 0.98 | ± | 0.12 | 0.32 | ± | 0.07 | 0.09 | ± | 0.03 | 0.14 | ± | 0.03 | 0.54 | ± | 0.04 | 1.03 | ± | 0.07 | 0.19 | ± | 0.03 | 0.29 | ± | 0.05 | 0.23 | ± | 0.08 |
| **Non-lobe WM** | 0.32 | ± | 0.02 | 0.21 | ± | 0.04 | 0.81 | ± | 0.11 | 0.19 | ± | 0.04 | 0.14 | ± | 0.02 | 0.17 | ± | 0.02 | 0.71 | ± | 0.06 | 1.31 | ± | 0.08 | 0.29 | ± | 0.04 | 0.27 | ± | 0.04 | 0.22 | ± | 0.07 |
| **Cortical GM (left)** | 0.24 | ± | 0.01 | 0.28 | ± | 0.04 | 1.18 | ± | 0.09 | 0.33 | ± | 0.06 | 0.12 | ± | 0.02 | 0.11 | ± | 0.01 | 0.73 | ± | 0.04 | 1.33 | ± | 0.09 | 0.26 | ± | 0.03 | 0.28 | ± | 0.04 | 0.33 | ± | 0.05 |
| **Cortical GM (right)** | 0.24 | ± | 0.01 | 0.27 | ± | 0.05 | 1.16 | ± | 0.10 | 0.32 | ± | 0.05 | 0.12 | ± | 0.01 | 0.11 | ± | 0.01 | 0.76 | ± | 0.04 | 1.32 | ± | 0.08 | 0.26 | ± | 0.03 | 0.26 | ± | 0.04 | 0.33 | ± | 0.04 |
| **Cortical GM (bilateral)** | 0.24 | ± | 0.01 | 0.27 | ± | 0.04 | 1.17 | ± | 0.09 | 0.32 | ± | 0.05 | 0.12 | ± | 0.01 | 0.11 | ± | 0.01 | 0.74 | ± | 0.04 | 1.33 | ± | 0.09 | 0.26 | ± | 0.03 | 0.27 | ± | 0.03 | 0.33 | ± | 0.04 |
| **Cortical GM+ subcortical WM (left)** | 0.25 | ± | 0.02 | 0.26 | ± | 0.04 | 1.12 | ± | 0.09 | 0.30 | ± | 0.06 | 0.12 | ± | 0.02 | 0.12 | ± | 0.01 | 0.72 | ± | 0.04 | 1.35 | ± | 0.09 | 0.26 | ± | 0.03 | 0.28 | ± | 0.04 | 0.31 | ± | 0.06 |
| **Cortical GM+ subcortical WM (right)** | 0.26 | ± | 0.01 | 0.26 | ± | 0.05 | 1.11 | ± | 0.10 | 0.29 | ± | 0.05 | 0.12 | ± | 0.01 | 0.12 | ± | 0.01 | 0.75 | ± | 0.04 | 1.33 | ± | 0.08 | 0.27 | ± | 0.03 | 0.26 | ± | 0.04 | 0.31 | ± | 0.04 |
| **Cortical GM+ subcortical WM (bilateral)** | 0.25 | ± | 0.02 | 0.26 | ± | 0.04 | 1.12 | ± | 0.09 | 0.30 | ± | 0.05 | 0.12 | ± | 0.01 | 0.12 | ± | 0.01 | 0.74 | ± | 0.04 | 1.34 | ± | 0.09 | 0.26 | ± | 0.03 | 0.27 | ± | 0.03 | 0.31 | ± | 0.04 |
| **Subcortical GM (left)** | 0.27 | ± | 0.02 | 0.21 | ± | 0.04 | 0.95 | ± | 0.12 | 0.28 | ± | 0.05 | 0.11 | ± | 0.03 | 0.16 | ± | 0.02 | 0.64 | ± | 0.07 | 1.11 | ± | 0.07 | 0.24 | ± | 0.04 | 0.29 | ± | 0.04 | 0.25 | ± | 0.09 |
| **Subcortical GM (right)** | 0.27 | ± | 0.02 | 0.26 | ± | 0.07 | 0.98 | ± | 0.13 | 0.32 | ± | 0.07 | 0.11 | ± | 0.02 | 0.18 | ± | 0.03 | 0.67 | ± | 0.07 | 1.07 | ± | 0.09 | 0.24 | ± | 0.04 | 0.32 | ± | 0.05 | 0.25 | ± | 0.08 |
| **Subcortical GM (bilateral)** | 0.27 | ± | 0.02 | 0.23 | ± | 0.04 | 0.96 | ± | 0.12 | 0.29 | ± | 0.06 | 0.11 | ± | 0.02 | 0.17 | ± | 0.02 | 0.66 | ± | 0.06 | 1.09 | ± | 0.07 | 0.24 | ± | 0.03 | 0.30 | ± | 0.04 | 0.25 | ± | 0.08 |
| **Auditory subcortex WM** | 0.27 | ± | 0.02 | 0.23 | ± | 0.06 | 1.17 | ± | 0.14 | 0.36 | ± | 0.09 | 0.11 | ± | 0.02 | 0.13 | ± | 0.02 | 0.73 | ± | 0.07 | 1.25 | ± | 0.11 | 0.22 | ± | 0.05 | 0.31 | ± | 0.04 | 0.29 | ± | 0.07 |
| **Auditory cortex GM** | 0.25 | ± | 0.02 | 0.25 | ± | 0.06 | 1.23 | ± | 0.13 | 0.39 | ± | 0.08 | 0.11 | ± | 0.02 | 0.13 | ± | 0.02 | 0.74 | ± | 0.06 | 1.23 | ± | 0.11 | 0.22 | ± | 0.05 | 0.31 | ± | 0.04 | 0.33 | ± | 0.07 |
| **Auditory cortex/subcortex GM+WM** | 0.26 | ± | 0.02 | 0.24 | ± | 0.06 | 1.20 | ± | 0.14 | 0.38 | ± | 0.08 | 0.11 | ± | 0.02 | 0.13 | ± | 0.02 | 0.73 | ± | 0.06 | 1.24 | ± | 0.11 | 0.22 | ± | 0.05 | 0.31 | ± | 0.04 | 0.31 | ± | 0.07 |
| **Occipital subcortex WM** | 0.25 | ± | 0.03 | 0.30 | ± | 0.10 | 1.04 | ± | 0.14 | 0.31 | ± | 0.07 | 0.13 | ± | 0.03 | 0.14 | ± | 0.03 | 0.70 | ± | 0.06 | 1.37 | ± | 0.17 | 0.27 | ± | 0.08 | 0.31 | ± | 0.06 | 0.29 | ± | 0.06 |
| **Occipital cortex GM** | 0.23 | ± | 0.02 | 0.33 | ± | 0.10 | 1.14 | ± | 0.13 | 0.35 | ± | 0.07 | 0.13 | ± | 0.03 | 0.13 | ± | 0.02 | 0.70 | ± | 0.05 | 1.35 | ± | 0.18 | 0.28 | ± | 0.07 | 0.31 | ± | 0.06 | 0.33 | ± | 0.06 |
| **Occipital cortex/subcortex GM+WM** | 0.24 | ± | 0.02 | 0.31 | ± | 0.10 | 1.08 | ± | 0.14 | 0.33 | ± | 0.07 | 0.13 | ± | 0.03 | 0.14 | ± | 0.02 | 0.70 | ± | 0.06 | 1.36 | ± | 0.17 | 0.27 | ± | 0.08 | 0.31 | ± | 0.06 | 0.31 | ± | 0.06 |
| **Temporal subcortex WM** | 0.28 | ± | 0.02 | 0.23 | ± | 0.06 | 1.08 | ± | 0.12 | 0.33 | ± | 0.07 | 0.12 | ± | 0.02 | 0.14 | ± | 0.02 | 0.70 | ± | 0.06 | 1.25 | ± | 0.11 | 0.25 | ± | 0.04 | 0.30 | ± | 0.04 | 0.28 | ± | 0.06 |
| **Temporal cortex/subcortex GM+WM** | 0.28 | ± | 0.02 | 0.24 | ± | 0.06 | 1.13 | ± | 0.12 | 0.36 | ± | 0.07 | 0.12 | ± | 0.02 | 0.13 | ± | 0.02 | 0.72 | ± | 0.06 | 1.25 | ± | 0.11 | 0.25 | ± | 0.04 | 0.30 | ± | 0.04 | 0.31 | ± | 0.06 |
| **Frontal subcortex WM** | 0.27 | ± | 0.02 | 0.23 | ± | 0.02 | 1.09 | ± | 0.08 | 0.27 | ± | 0.06 | 0.11 | ± | 0.01 | 0.13 | ± | 0.02 | 0.70 | ± | 0.04 | 1.35 | ± | 0.08 | 0.26 | ± | 0.03 | 0.26 | ± | 0.04 | 0.29 | ± | 0.05 |
| **Frontal cortex GM** | 0.25 | ± | 0.02 | 0.26 | ± | 0.03 | 1.19 | ± | 0.09 | 0.32 | ± | 0.06 | 0.11 | ± | 0.01 | 0.12 | ± | 0.02 | 0.75 | ± | 0.04 | 1.36 | ± | 0.08 | 0.26 | ± | 0.03 | 0.28 | ± | 0.04 | 0.33 | ± | 0.05 |
| **Frontal cortex/subcortex GM+WM** | 0.26 | ± | 0.02 | 0.24 | ± | 0.03 | 1.13 | ± | 0.08 | 0.29 | ± | 0.06 | 0.11 | ± | 0.01 | 0.12 | ± | 0.02 | 0.72 | ± | 0.04 | 1.35 | ± | 0.08 | 0.26 | ± | 0.03 | 0.27 | ± | 0.04 | 0.31 | ± | 0.05 |
| **Visual cortex GM** | 0.21 | ± | 0.02 | 0.31 | ± | 0.12 | 1.12 | ± | 0.15 | 0.33 | ± | 0.09 | 0.13 | ± | 0.03 | 0.13 | ± | 0.03 | 0.68 | ± | 0.06 | 1.42 | ± | 0.20 | 0.29 | ± | 0.08 | 0.33 | ± | 0.06 | 0.32 | ± | 0.07 |
| **Visual cortex/subcortex GM+WM** | 0.22 | ± | 0.02 | 0.30 | ± | 0.12 | 1.06 | ± | 0.16 | 0.32 | ± | 0.08 | 0.13 | ± | 0.03 | 0.14 | ± | 0.03 | 0.68 | ± | 0.06 | 1.43 | ± | 0.19 | 0.29 | ± | 0.08 | 0.32 | ± | 0.06 | 0.30 | ± | 0.07 |
| **Primary somatosensory cortex GM** | 0.22 | ± | 0.01 | 0.23 | ± | 0.05 | 1.11 | ± | 0.10 | 0.29 | ± | 0.08 | 0.12 | ± | 0.02 | 0.08 | ± | 0.02 | 0.71 | ± | 0.05 | 1.31 | ± | 0.12 | 0.27 | ± | 0.04 | 0.24 | ± | 0.04 | 0.33 | ± | 0.05 |
| **Pallidum** | 0.24 | ± | 0.02 | 0.25 | ± | 0.08 | 0.90 | ± | 0.17 | 0.30 | ± | 0.10 | 0.08 | ± | 0.03 | 0.17 | ± | 0.03 | 0.48 | ± | 0.07 | 0.94 | ± | 0.13 | 0.21 | ± | 0.08 | 0.30 | ± | 0.08 | 0.20 | ± | 0.10 |
| **Hippocampus** | 0.30 | ± | 0.02 | 0.25 | ± | 0.10 | 0.94 | ± | 0.13 | 0.32 | ± | 0.08 | 0.14 | ± | 0.03 | 0.20 | ± | 0.03 | 0.79 | ± | 0.09 | 1.00 | ± | 0.13 | 0.24 | ± | 0.07 | 0.28 | ± | 0.05 | 0.30 | ± | 0.07 |
| **Corpus callosum** | 0.35 | ± | 0.03 | 0.29 | ± | 0.09 | 1.13 | ± | 0.28 | 0.25 | ± | 0.11 | 0.17 | ± | 0.04 | 0.21 | ± | 0.04 | 0.89 | ± | 0.12 | 1.64 | ± | 0.25 | 0.33 | ± | 0.09 | 0.36 | ± | 0.09 | 0.27 | ± | 0.10 |
| **Mean** | 0.26 |  |  | 0.25 |  |  | 1.08 |  |  | 0.29 |  |  | 0.12 |  |  | 0.13 |  |  | 0.72 |  |  | 1.30 |  |  | 0.26 |  |  | 0.28 |  |  | 0.29 |  |  |
| **Min** | 0.21 |  |  | 0.19 |  |  | 0.81 |  |  | 0.19 |  |  | 0.08 |  |  | 0.08 |  |  | 0.48 |  |  | 0.94 |  |  | 0.19 |  |  | 0.21 |  |  | 0.20 |  |  |
| **Max** | 0.35 |  |  | 0.33 |  |  | 1.23 |  |  | 0.39 |  |  | 0.17 |  |  | 0.21 |  |  | 0.89 |  |  | 1.64 |  |  | 0.33 |  |  | 0.36 |  |  | 0.33 |  |  |

Supplementary Table 1: Mean ratios to tCr and their standard deviations for all other metabolites, based on the overall ROI concentration estimates.

| **Supplementary Table 2 - Inter-subject CVs for ratios to tCr per ROI for all metabolites** | | | | | | | | | | | |
| --- | --- | --- | --- | --- | --- | --- | --- | --- | --- | --- | --- |
| **ROI** | **tCho** | **GABA** | **Glu** | **Gln** | **Gly** | **GSH** | **mIns** | **NAA** | **NAAG** | **Ser** | **Tau** |
| **Subcortical WM (left)** | 7% | 16% | 8% | 22% | 13% | 12% | 5% | 7% | 11% | 14% | 20% |
| **Subcortical WM (right)** | 6% | 18% | 9% | 18% | 11% | 10% | 6% | 7% | 12% | 14% | 13% |
| **Subcortical WM (bilateral)** | 6% | 16% | 8% | 19% | 11% | 10% | 5% | 6% | 11% | 13% | 16% |
| **Motor subcortex WM** | 7% | 18% | 9% | 32% | 9% | 16% | 5% | 7% | 9% | 19% | 15% |
| **Motor cortex GM** | 7% | 17% | 8% | 26% | 11% | 15% | 6% | 7% | 9% | 18% | 13% |
| **Motor cortex/subcortex GM+WM** | 7% | 17% | 8% | 28% | 9% | 15% | 5% | 7% | 9% | 18% | 14% |
| **Parietal subcortex WM** | 6% | 28% | 8% | 22% | 15% | 13% | 6% | 6% | 10% | 18% | 15% |
| **Parietal cortex GM** | 6% | 26% | 7% | 21% | 18% | 13% | 6% | 6% | 10% | 19% | 12% |
| **Parietal cortex/subcortex GM+WM** | 6% | 27% | 8% | 21% | 16% | 13% | 6% | 6% | 10% | 19% | 14% |
| **Cingulate subcortex WM** | 8% | 26% | 15% | 25% | 13% | 17% | 13% | 8% | 11% | 14% | 21% |
| **Cingulate cortex GM** | 7% | 22% | 12% | 22% | 12% | 20% | 10% | 6% | 14% | 15% | 16% |
| **Cingulate cortex/subcortex GM+WM** | 7% | 24% | 13% | 22% | 13% | 17% | 11% | 7% | 12% | 14% | 18% |
| **Visual subcortex WM** | 11% | 41% | 17% | 28% | 24% | 23% | 9% | 14% | 29% | 21% | 24% |
| **Primary somatosensory subcortex WM** | 5% | 21% | 9% | 27% | 14% | 17% | 7% | 8% | 13% | 15% | 16% |
| **Primary somatosensory cortex/subcortex GM+WM** | 5% | 20% | 9% | 27% | 13% | 17% | 7% | 9% | 13% | 15% | 16% |
| **Thalamus** | 10% | 34% | 22% | 38% | 25% | 24% | 19% | 12% | 23% | 27% | 41% |
| **Putamen** | 5% | 27% | 12% | 21% | 35% | 18% | 8% | 7% | 17% | 18% | 36% |
| **Non-lobe WM** | 7% | 21% | 13% | 20% | 16% | 12% | 9% | 6% | 12% | 14% | 31% |
| **Cortical GM (left)** | 6% | 16% | 7% | 19% | 14% | 12% | 5% | 7% | 12% | 13% | 16% |
| **Cortical GM (right)** | 6% | 18% | 8% | 16% | 10% | 11% | 6% | 6% | 12% | 14% | 11% |
| **Cortical GM (bilateral)** | 6% | 16% | 8% | 17% | 11% | 10% | 5% | 6% | 12% | 13% | 13% |
| **Cortical GM+ subcortical WM (left)** | 6% | 16% | 8% | 20% | 13% | 11% | 5% | 7% | 11% | 13% | 18% |
| **Cortical GM+ subcortical WM (right)** | 6% | 18% | 9% | 17% | 10% | 10% | 6% | 6% | 11% | 14% | 12% |
| **Cortical GM+ subcortical WM (bilateral)** | 6% | 16% | 8% | 17% | 11% | 10% | 5% | 6% | 11% | 13% | 15% |
| **Subcortical GM (left)** | 6% | 17% | 13% | 19% | 23% | 15% | 11% | 6% | 15% | 15% | 37% |
| **Subcortical GM (right)** | 7% | 25% | 13% | 22% | 21% | 16% | 11% | 9% | 18% | 16% | 31% |
| **Subcortical GM (bilateral)** | 6% | 19% | 12% | 19% | 21% | 13% | 10% | 6% | 14% | 14% | 33% |
| **Auditory subcortex WM** | 8% | 27% | 12% | 24% | 22% | 18% | 9% | 9% | 23% | 13% | 25% |
| **Auditory cortex GM** | 7% | 22% | 11% | 19% | 21% | 19% | 9% | 9% | 22% | 14% | 20% |
| **Auditory cortex/subcortex GM+WM** | 7% | 25% | 11% | 21% | 21% | 18% | 9% | 9% | 23% | 13% | 22% |
| **Occipital subcortex WM** | 10% | 33% | 13% | 23% | 22% | 19% | 8% | 13% | 28% | 20% | 21% |
| **Occipital cortex GM** | 9% | 31% | 12% | 20% | 21% | 17% | 7% | 13% | 27% | 19% | 17% |
| **Occipital cortex/subcortex GM+WM** | 10% | 32% | 13% | 22% | 21% | 18% | 8% | 13% | 28% | 19% | 19% |
| **Temporal subcortex WM** | 7% | 25% | 11% | 21% | 15% | 12% | 8% | 8% | 16% | 14% | 22% |
| **Temporal cortex/subcortex GM+WM** | 7% | 25% | 11% | 19% | 15% | 13% | 8% | 9% | 16% | 13% | 20% |
| **Frontal subcortex WM** | 6% | 10% | 7% | 22% | 13% | 17% | 6% | 6% | 12% | 16% | 17% |
| **Frontal cortex GM** | 6% | 12% | 7% | 19% | 13% | 17% | 5% | 6% | 11% | 15% | 15% |
| **Frontal cortex/subcortex GM+WM** | 6% | 11% | 7% | 20% | 13% | 17% | 5% | 6% | 11% | 15% | 16% |
| **Visual cortex GM** | 10% | 39% | 14% | 26% | 22% | 23% | 8% | 14% | 29% | 19% | 22% |
| **Visual cortex/subcortex GM+WM** | 10% | 40% | 15% | 27% | 23% | 22% | 9% | 14% | 29% | 20% | 23% |
| **Primary somatosensory cortex GM** | 5% | 20% | 9% | 26% | 13% | 19% | 7% | 9% | 13% | 15% | 16% |
| **Pallidum** | 10% | 32% | 19% | 32% | 41% | 18% | 14% | 14% | 39% | 28% | 50% |
| **Hippocampus** | 7% | 38% | 13% | 26% | 21% | 17% | 12% | 13% | 30% | 17% | 24% |
| **Corpus callosum** | 9% | 32% | 25% | 43% | 26% | 21% | 14% | 15% | 26% | 24% | 38% |

Supplementary Table 2: Inter-subject CVs for ratios to tCr per ROI, with high similarity to concentration estimate CVs, as described in Tbl.5.

| **Supplementary Table 3 - Mean concentration estimates of all quantified metabolites per ROI [mM]** | | | | | | | | | | | | | | | | | | | | | | | | | | | | | | | | | | | | |
| --- | --- | --- | --- | --- | --- | --- | --- | --- | --- | --- | --- | --- | --- | --- | --- | --- | --- | --- | --- | --- | --- | --- | --- | --- | --- | --- | --- | --- | --- | --- | --- | --- | --- | --- | --- | --- |
| **ROI** | **tCho** | | | **tCr** | | | **GABA** | | | **Glu** | | | **Gln** | | | **Gly** | | | **GSH** | | | **mIns** | | | **NAA** | | | **NAAG** | | | **Ser** | | | **Tau** | | |
| **Subcortical WM (left)** | 1.89 | ± | 0.76 | 7.24 | ± | 2.72 | 1.75 | ± | 1.24 | 7.68 | ± | 3.35 | 2.12 | ± | 1.58 | 0.92 | ± | 0.62 | 0.91 | ± | 0.68 | 5.15 | ± | 1.92 | 9.78 | ± | 3.77 | 1.93 | ± | 1.11 | 1.94 | ± | 1.23 | 2.11 | ± | 1.34 |
| **Subcortical WM (right)** | 1.95 | ± | 0.71 | 7.25 | ± | 2.50 | 1.76 | ± | 1.21 | 7.67 | ± | 3.23 | 1.99 | ± | 1.47 | 0.89 | ± | 0.55 | 0.90 | ± | 0.62 | 5.35 | ± | 1.77 | 9.67 | ± | 3.44 | 1.95 | ± | 1.09 | 1.83 | ± | 1.17 | 2.09 | ± | 1.17 |
| **Subcortical WM (bilateral)** | 1.92 | ± | 0.73 | 7.24 | ± | 2.62 | 1.75 | ± | 1.23 | 7.67 | ± | 3.29 | 2.05 | ± | 1.53 | 0.90 | ± | 0.59 | 0.91 | ± | 0.65 | 5.24 | ± | 1.85 | 9.73 | ± | 3.61 | 1.94 | ± | 1.10 | 1.89 | ± | 1.21 | 2.10 | ± | 1.27 |
| **Motor subcortex WM** | 2.00 | ± | 0.47 | 7.96 | ± | 1.61 | 1.73 | ± | 0.95 | 7.90 | ± | 2.25 | 1.70 | ± | 1.24 | 1.06 | ± | 0.47 | 0.74 | ± | 0.42 | 5.45 | ± | 1.15 | 10.72 | ± | 2.11 | 2.51 | ± | 0.88 | 1.71 | ± | 0.83 | 2.30 | ± | 0.94 |
| **Motor cortex GM** | 1.71 | ± | 0.50 | 7.55 | ± | 2.06 | 1.84 | ± | 1.08 | 8.42 | ± | 2.58 | 2.05 | ± | 1.39 | 0.93 | ± | 0.53 | 0.61 | ± | 0.43 | 5.39 | ± | 1.72 | 10.15 | ± | 2.79 | 2.18 | ± | 0.96 | 1.78 | ± | 0.95 | 2.51 | ± | 1.14 |
| **Motor cortex/subcortex GM+WM** | 1.87 | ± | 0.50 | 7.78 | ± | 1.83 | 1.78 | ± | 1.01 | 8.13 | ± | 2.41 | 1.85 | ± | 1.32 | 1.00 | ± | 0.50 | 0.68 | ± | 0.43 | 5.42 | ± | 1.43 | 10.48 | ± | 2.44 | 2.37 | ± | 0.93 | 1.74 | ± | 0.88 | 2.39 | ± | 1.04 |
| **Parietal subcortex WM** | 1.84 | ± | 0.51 | 7.51 | ± | 2.00 | 1.85 | ± | 1.14 | 7.64 | ± | 2.61 | 1.90 | ± | 1.25 | 0.97 | ± | 0.45 | 0.80 | ± | 0.48 | 5.54 | ± | 1.38 | 10.21 | ± | 2.66 | 2.15 | ± | 0.91 | 1.68 | ± | 0.85 | 2.14 | ± | 0.95 |
| **Parietal cortex GM** | 1.64 | ± | 0.50 | 7.57 | ± | 2.23 | 2.06 | ± | 1.22 | 8.55 | ± | 2.81 | 2.24 | ± | 1.33 | 0.92 | ± | 0.44 | 0.69 | ± | 0.46 | 5.61 | ± | 1.63 | 10.09 | ± | 3.02 | 2.02 | ± | 0.95 | 1.78 | ± | 0.94 | 2.41 | ± | 1.00 |
| **Parietal cortex/subcortex GM+WM** | 1.74 | ± | 0.52 | 7.54 | ± | 2.11 | 1.95 | ± | 1.19 | 8.07 | ± | 2.74 | 2.06 | ± | 1.30 | 0.94 | ± | 0.45 | 0.75 | ± | 0.47 | 5.58 | ± | 1.50 | 10.15 | ± | 2.83 | 2.08 | ± | 0.93 | 1.72 | ± | 0.89 | 2.27 | ± | 0.98 |
| **Cingulate subcortex WM** | 2.32 | ± | 0.81 | 7.55 | ± | 2.75 | 1.93 | ± | 1.25 | 8.06 | ± | 3.70 | 1.83 | ± | 1.52 | 1.06 | ± | 0.70 | 1.22 | ± | 0.73 | 5.99 | ± | 2.01 | 10.55 | ± | 3.57 | 2.06 | ± | 1.08 | 2.26 | ± | 1.38 | 2.09 | ± | 1.47 |
| **Cingulate cortex GM** | 2.22 | ± | 0.74 | 8.45 | ± | 2.62 | 2.36 | ± | 1.28 | 10.14 | ± | 3.57 | 2.47 | ± | 1.52 | 1.00 | ± | 0.63 | 1.05 | ± | 0.72 | 6.60 | ± | 2.05 | 10.86 | ± | 3.41 | 2.06 | ± | 1.04 | 2.40 | ± | 1.43 | 2.81 | ± | 1.47 |
| **Cingulate cortex/subcortex GM+WM** | 2.28 | ± | 0.78 | 7.94 | ± | 2.73 | 2.12 | ± | 1.28 | 8.98 | ± | 3.79 | 2.10 | ± | 1.56 | 1.03 | ± | 0.67 | 1.14 | ± | 0.73 | 6.26 | ± | 2.05 | 10.68 | ± | 3.51 | 2.06 | ± | 1.07 | 2.32 | ± | 1.40 | 2.41 | ± | 1.52 |
| **Visual subcortex WM** | 1.54 | ± | 0.57 | 6.74 | ± | 2.44 | 1.93 | ± | 1.51 | 6.64 | ± | 3.18 | 2.10 | ± | 1.58 | 0.96 | ± | 0.59 | 0.92 | ± | 0.63 | 4.60 | ± | 1.62 | 9.31 | ± | 3.69 | 1.95 | ± | 1.26 | 2.09 | ± | 1.19 | 1.88 | ± | 1.05 |
| **Primary somatosensory subcortex WM** | 1.73 | ± | 0.50 | 7.57 | ± | 1.93 | 1.63 | ± | 0.97 | 7.88 | ± | 2.27 | 2.07 | ± | 1.32 | 0.90 | ± | 0.47 | 0.66 | ± | 0.46 | 5.21 | ± | 1.27 | 10.06 | ± | 2.57 | 2.17 | ± | 0.92 | 1.74 | ± | 0.88 | 2.23 | ± | 1.01 |
| **Primary somatosensory cortex/subcortex GM+WM** | 1.65 | ± | 0.52 | 7.40 | ± | 2.14 | 1.62 | ± | 0.97 | 7.90 | ± | 2.48 | 2.12 | ± | 1.36 | 0.89 | ± | 0.47 | 0.62 | ± | 0.46 | 5.16 | ± | 1.46 | 9.73 | ± | 2.84 | 2.08 | ± | 0.96 | 1.73 | ± | 0.91 | 2.30 | ± | 1.04 |
| **Thalamus** | 2.40 | ± | 0.83 | 8.81 | ± | 3.07 | 2.47 | ± | 1.48 | 9.18 | ± | 4.15 | 2.52 | ± | 2.13 | 1.16 | ± | 0.95 | 1.37 | ± | 0.82 | 6.24 | ± | 2.21 | 10.60 | ± | 4.00 | 2.58 | ± | 1.55 | 2.69 | ± | 1.58 | 2.41 | ± | 2.08 |
| **Putamen** | 2.27 | ± | 0.86 | 9.36 | ± | 3.32 | 1.83 | ± | 1.26 | 9.20 | ± | 3.59 | 3.13 | ± | 1.70 | 0.87 | ± | 0.84 | 1.33 | ± | 0.83 | 5.09 | ± | 2.04 | 9.58 | ± | 3.77 | 1.83 | ± | 1.13 | 2.80 | ± | 1.58 | 2.26 | ± | 1.78 |
| **Non-lobe WM** | 2.42 | ± | 0.76 | 7.59 | ± | 2.53 | 1.60 | ± | 1.19 | 6.18 | ± | 2.75 | 1.59 | ± | 1.35 | 1.05 | ± | 0.69 | 1.32 | ± | 0.71 | 5.34 | ± | 1.71 | 9.88 | ± | 3.14 | 2.21 | ± | 1.18 | 2.10 | ± | 1.30 | 1.77 | ± | 1.33 |
| **Cortical GM (left)** | 1.64 | ± | 0.76 | 6.92 | ± | 3.06 | 1.90 | ± | 1.43 | 8.13 | ± | 3.99 | 2.35 | ± | 1.70 | 0.84 | ± | 0.65 | 0.79 | ± | 0.67 | 5.06 | ± | 2.28 | 9.16 | ± | 4.47 | 1.80 | ± | 1.20 | 1.96 | ± | 1.30 | 2.31 | ± | 1.44 |
| **Cortical GM (right)** | 1.75 | ± | 0.64 | 7.25 | ± | 2.58 | 1.96 | ± | 1.37 | 8.35 | ± | 3.35 | 2.32 | ± | 1.49 | 0.85 | ± | 0.53 | 0.80 | ± | 0.65 | 5.49 | ± | 1.91 | 9.52 | ± | 3.49 | 1.89 | ± | 1.10 | 1.87 | ± | 1.36 | 2.43 | ± | 1.23 |
| **Cortical GM (bilateral)** | 1.69 | ± | 0.71 | 7.07 | ± | 2.85 | 1.92 | ± | 1.40 | 8.23 | ± | 3.70 | 2.33 | ± | 1.60 | 0.84 | ± | 0.59 | 0.79 | ± | 0.66 | 5.26 | ± | 2.12 | 9.33 | ± | 4.05 | 1.84 | ± | 1.15 | 1.91 | ± | 1.33 | 2.36 | ± | 1.35 |
| **Cortical GM+ subcortical WM (left)** | 1.78 | ± | 0.77 | 7.09 | ± | 2.89 | 1.82 | ± | 1.34 | 7.89 | ± | 3.67 | 2.22 | ± | 1.64 | 0.88 | ± | 0.64 | 0.85 | ± | 0.68 | 5.11 | ± | 2.10 | 9.49 | ± | 4.14 | 1.87 | ± | 1.16 | 1.95 | ± | 1.26 | 2.21 | ± | 1.40 |
| **Cortical GM+ subcortical WM (right)** | 1.86 | ± | 0.69 | 7.25 | ± | 2.54 | 1.85 | ± | 1.29 | 7.98 | ± | 3.31 | 2.14 | ± | 1.49 | 0.87 | ± | 0.54 | 0.85 | ± | 0.63 | 5.41 | ± | 1.84 | 9.60 | ± | 3.47 | 1.92 | ± | 1.10 | 1.85 | ± | 1.26 | 2.24 | ± | 1.21 |
| **Cortical GM+ subcortical WM (bilateral)** | 1.81 | ± | 0.73 | 7.16 | ± | 2.73 | 1.83 | ± | 1.32 | 7.93 | ± | 3.50 | 2.18 | ± | 1.57 | 0.87 | ± | 0.59 | 0.85 | ± | 0.66 | 5.25 | ± | 1.98 | 9.54 | ± | 3.83 | 1.89 | ± | 1.13 | 1.90 | ± | 1.27 | 2.22 | ± | 1.31 |
| **Subcortical GM (left)** | 2.32 | ± | 0.95 | 8.61 | ± | 3.64 | 1.89 | ± | 1.41 | 8.20 | ± | 4.06 | 2.42 | ± | 1.90 | 0.96 | ± | 0.91 | 1.43 | ± | 0.94 | 5.58 | ± | 2.48 | 9.50 | ± | 4.21 | 2.13 | ± | 1.47 | 2.53 | ± | 1.74 | 2.27 | ± | 2.13 |
| **Subcortical GM (right)** | 2.02 | ± | 0.97 | 7.40 | ± | 3.68 | 1.98 | ± | 1.57 | 7.24 | ± | 4.16 | 2.40 | ± | 1.89 | 0.89 | ± | 0.85 | 1.33 | ± | 0.90 | 4.99 | ± | 2.48 | 7.83 | ± | 4.32 | 1.82 | ± | 1.40 | 2.36 | ± | 1.64 | 1.90 | ± | 1.68 |
| **Subcortical GM (bilateral)** | 2.17 | ± | 0.97 | 7.99 | ± | 3.72 | 1.93 | ± | 1.48 | 7.72 | ± | 4.15 | 2.40 | ± | 1.90 | 0.92 | ± | 0.88 | 1.37 | ± | 0.93 | 5.28 | ± | 2.51 | 8.66 | ± | 4.35 | 1.95 | ± | 1.45 | 2.44 | ± | 1.69 | 2.07 | ± | 1.94 |
| **Auditory subcortex WM** | 1.91 | ± | 0.72 | 7.13 | ± | 2.48 | 1.65 | ± | 1.19 | 8.14 | ± | 3.23 | 2.62 | ± | 1.59 | 0.79 | ± | 0.62 | 0.95 | ± | 0.68 | 5.13 | ± | 1.79 | 8.81 | ± | 3.45 | 1.53 | ± | 1.08 | 2.18 | ± | 1.35 | 2.07 | ± | 1.39 |
| **Auditory cortex GM** | 1.67 | ± | 0.69 | 6.69 | ± | 2.60 | 1.64 | ± | 1.20 | 8.10 | ± | 3.43 | 2.64 | ± | 1.60 | 0.74 | ± | 0.60 | 0.83 | ± | 0.65 | 4.88 | ± | 1.95 | 8.13 | ± | 3.56 | 1.45 | ± | 1.07 | 2.05 | ± | 1.34 | 2.22 | ± | 1.44 |
| **Auditory cortex/subcortex GM+WM** | 1.78 | ± | 0.71 | 6.89 | ± | 2.56 | 1.64 | ± | 1.19 | 8.11 | ± | 3.34 | 2.63 | ± | 1.59 | 0.77 | ± | 0.61 | 0.89 | ± | 0.67 | 4.99 | ± | 1.88 | 8.44 | ± | 3.53 | 1.48 | ± | 1.07 | 2.11 | ± | 1.34 | 2.15 | ± | 1.42 |
| **Occipital subcortex WM** | 1.61 | ± | 0.68 | 6.61 | ± | 2.66 | 1.96 | ± | 1.53 | 6.68 | ± | 3.35 | 2.11 | ± | 1.62 | 0.90 | ± | 0.61 | 0.91 | ± | 0.65 | 4.59 | ± | 1.79 | 8.83 | ± | 3.91 | 1.79 | ± | 1.25 | 2.00 | ± | 1.22 | 1.87 | ± | 1.15 |
| **Occipital cortex GM** | 1.46 | ± | 0.65 | 6.55 | ± | 2.92 | 2.07 | ± | 1.73 | 7.26 | ± | 3.72 | 2.30 | ± | 1.73 | 0.84 | ± | 0.59 | 0.82 | ± | 0.74 | 4.57 | ± | 2.00 | 8.58 | ± | 4.31 | 1.77 | ± | 1.33 | 1.99 | ± | 1.29 | 2.11 | ± | 1.28 |
| **Occipital cortex/subcortex GM+WM** | 1.54 | ± | 0.67 | 6.58 | ± | 2.79 | 2.01 | ± | 1.64 | 6.95 | ± | 3.55 | 2.19 | ± | 1.67 | 0.87 | ± | 0.60 | 0.87 | ± | 0.70 | 4.58 | ± | 1.89 | 8.71 | ± | 4.12 | 1.78 | ± | 1.29 | 1.99 | ± | 1.25 | 1.98 | ± | 1.22 |
| **Temporal subcortex WM** | 1.95 | ± | 0.89 | 6.89 | ± | 3.17 | 1.55 | ± | 1.19 | 7.30 | ± | 3.68 | 2.32 | ± | 1.64 | 0.83 | ± | 0.65 | 0.94 | ± | 0.69 | 4.83 | ± | 2.15 | 8.56 | ± | 4.06 | 1.66 | ± | 1.14 | 2.00 | ± | 1.34 | 1.97 | ± | 1.48 |
| **Temporal cortex/subcortex GM+WM** | 1.84 | ± | 0.87 | 6.74 | ± | 3.19 | 1.58 | ± | 1.29 | 7.47 | ± | 3.91 | 2.42 | ± | 1.66 | 0.81 | ± | 0.68 | 0.88 | ± | 0.71 | 4.82 | ± | 2.21 | 8.33 | ± | 4.63 | 1.63 | ± | 1.15 | 1.99 | ± | 1.35 | 2.08 | ± | 1.51 |
| **Frontal subcortex WM** | 1.99 | ± | 0.79 | 7.39 | ± | 2.69 | 1.71 | ± | 1.13 | 7.99 | ± | 3.41 | 2.09 | ± | 1.57 | 0.87 | ± | 0.61 | 0.95 | ± | 0.73 | 5.20 | ± | 1.92 | 9.91 | ± | 3.81 | 1.93 | ± | 1.11 | 1.93 | ± | 1.32 | 2.17 | ± | 1.34 |
| **Frontal cortex GM** | 1.74 | ± | 0.79 | 6.98 | ± | 3.01 | 1.84 | ± | 1.39 | 8.29 | ± | 3.98 | 2.29 | ± | 1.65 | 0.78 | ± | 0.64 | 0.87 | ± | 0.76 | 5.22 | ± | 2.29 | 9.44 | ± | 3.98 | 1.85 | ± | 1.19 | 1.96 | ± | 1.58 | 2.39 | ± | 1.50 |
| **Frontal cortex/subcortex GM+WM** | 1.88 | ± | 0.80 | 7.21 | ± | 2.84 | 1.76 | ± | 1.25 | 8.12 | ± | 3.67 | 2.18 | ± | 1.61 | 0.83 | ± | 0.63 | 0.92 | ± | 0.74 | 5.21 | ± | 2.09 | 9.70 | ± | 3.89 | 1.90 | ± | 1.15 | 1.94 | ± | 1.44 | 2.27 | ± | 1.42 |
| **Visual cortex GM** | 1.37 | ± | 0.55 | 6.74 | ± | 2.72 | 1.94 | ± | 1.55 | 7.28 | ± | 3.46 | 2.29 | ± | 1.68 | 0.90 | ± | 0.57 | 0.81 | ± | 0.62 | 4.53 | ± | 1.81 | 9.17 | ± | 3.99 | 1.95 | ± | 1.36 | 2.15 | ± | 1.29 | 2.08 | ± | 1.17 |
| **Visual cortex/subcortex GM+WM** | 1.46 | ± | 0.57 | 6.73 | ± | 2.57 | 1.93 | ± | 1.53 | 6.90 | ± | 3.32 | 2.16 | ± | 1.62 | 0.93 | ± | 0.59 | 0.87 | ± | 0.62 | 4.56 | ± | 1.71 | 9.23 | ± | 3.83 | 1.94 | ± | 1.31 | 2.11 | ± | 1.23 | 1.96 | ± | 1.11 |
| **Primary somatosensory cortex GM** | 1.57 | ± | 0.53 | 7.22 | ± | 2.33 | 1.62 | ± | 0.97 | 7.91 | ± | 2.69 | 2.19 | ± | 1.39 | 0.87 | ± | 0.48 | 0.59 | ± | 0.46 | 5.11 | ± | 1.63 | 9.37 | ± | 3.06 | 1.97 | ± | 0.98 | 1.72 | ± | 0.95 | 2.37 | ± | 1.07 |
| **Pallidum** | 2.11 | ± | 0.94 | 8.88 | ± | 3.75 | 2.41 | ± | 1.71 | 8.18 | ± | 4.30 | 2.89 | ± | 2.09 | 0.91 | ± | 0.94 | 1.63 | ± | 0.92 | 4.31 | ± | 2.03 | 8.43 | ± | 4.06 | 1.95 | ± | 1.43 | 2.85 | ± | 1.84 | 2.01 | ± | 2.02 |
| **Hippocampus** | 2.18 | ± | 1.01 | 7.37 | ± | 3.35 | 1.81 | ± | 1.55 | 6.83 | ± | 3.67 | 2.35 | ± | 1.72 | 1.07 | ± | 0.86 | 1.50 | ± | 0.97 | 5.78 | ± | 2.74 | 7.12 | ± | 3.73 | 1.80 | ± | 1.42 | 2.06 | ± | 1.56 | 2.26 | ± | 1.82 |
| **Corpus callosum** | 2.07 | ± | 0.88 | 5.93 | ± | 2.62 | 1.77 | ± | 1.35 | 6.54 | ± | 3.46 | 1.84 | ± | 1.73 | 1.10 | ± | 0.89 | 1.27 | ± | 0.75 | 5.25 | ± | 2.31 | 9.50 | ± | 4.28 | 2.01 | ± | 1.29 | 2.18 | ± | 1.41 | 1.86 | ± | 1.62 |
| **Mean** | 1.88 |  |  | 7.37 |  |  | 1.86 |  |  | 7.85 |  |  | 2.23 |  |  | 0.91 |  |  | 0.96 |  |  | 5.23 |  |  | 9.43 |  |  | 1.95 |  |  | 2.05 |  |  | 2.19 |  |  |
| **Min** | 1.37 |  |  | 5.93 |  |  | 1.55 |  |  | 6.18 |  |  | 1.59 |  |  | 0.74 |  |  | 0.59 |  |  | 4.31 |  |  | 7.12 |  |  | 1.45 |  |  | 1.68 |  |  | 1.77 |  |  |
| **Max** | 2.42 |  |  | 9.36 |  |  | 2.47 |  |  | 10.14 |  |  | 3.13 |  |  | 1.16 |  |  | 1.63 |  |  | 6.60 |  |  | 10.86 |  |  | 2.58 |  |  | 2.85 |  |  | 2.81 |  |  |

Supplementary Table 3: Mean concentration estimates per ROI [mM] and their standard deviations for all quantified metabolites in all qualified ROIs.

| **Table 6 - Inter-subject CVs for concentration estimates of all metabolites per ROI** | | | | | | | | | | | | |
| --- | --- | --- | --- | --- | --- | --- | --- | --- | --- | --- | --- | --- |
| **ROI** | **tCho** | **tCr** | **GABA** | **Glu** | **Gln** | **Gly** | **GSH** | **mIns** | **NAA** | **NAAG** | **Ser** | **Tau** |
| **Subcortical WM (left)** | 7% | 7% | 13% | 7% | 21% | 14% | 11% | 8% | 7% | 10% | 12% | 22% |
| **Subcortical WM (right)** | 6% | 6% | 15% | 8% | 17% | 10% | 11% | 6% | 6% | 11% | 13% | 14% |
| **Subcortical WM (bilateral)** | 6% | 6% | 13% | 7% | 18% | 11% | 10% | 7% | 6% | 11% | 12% | 17% |
| **Motor subcortex WM** | 8% | 7% | 16% | 7% | 31% | 10% | 19% | 7% | 7% | 11% | 18% | 17% |
| **Motor cortex GM** | 6% | 8% | 16% | 8% | 25% | 12% | 15% | 8% | 9% | 13% | 15% | 16% |
| **Motor cortex/subcortex GM+WM** | 7% | 7% | 16% | 7% | 28% | 11% | 17% | 8% | 8% | 12% | 16% | 16% |
| **Parietal subcortex WM** | 11% | 10% | 22% | 10% | 22% | 13% | 17% | 10% | 10% | 13% | 21% | 18% |
| **Parietal cortex GM** | 10% | 10% | 20% | 11% | 21% | 14% | 17% | 10% | 11% | 15% | 22% | 17% |
| **Parietal cortex/subcortex GM+WM** | 10% | 10% | 21% | 11% | 21% | 13% | 17% | 10% | 11% | 14% | 21% | 17% |
| **Cingulate subcortex WM** | 9% | 10% | 21% | 12% | 23% | 19% | 17% | 10% | 9% | 15% | 16% | 25% |
| **Cingulate cortex GM** | 9% | 10% | 19% | 12% | 20% | 16% | 21% | 10% | 10% | 18% | 18% | 19% |
| **Cingulate cortex/subcortex GM+WM** | 9% | 10% | 20% | 12% | 20% | 17% | 18% | 10% | 9% | 15% | 16% | 22% |
| **Visual subcortex WM** | 13% | 15% | 30% | 14% | 27% | 21% | 16% | 14% | 8% | 22% | 17% | 15% |
| **Primary somatosensory subcortex WM** | 8% | 9% | 20% | 8% | 30% | 14% | 19% | 10% | 8% | 14% | 16% | 18% |
| **Primary somatosensory cortex/subcortex GM+WM** | 8% | 9% | 19% | 8% | 30% | 14% | 18% | 10% | 9% | 14% | 16% | 18% |
| **Thalamus** | 19% | 20% | 28% | 21% | 35% | 31% | 24% | 19% | 16% | 27% | 23% | 48% |
| **Putamen** | 9% | 9% | 26% | 15% | 25% | 33% | 19% | 13% | 11% | 20% | 21% | 35% |
| **Non-lobe WM** | 10% | 7% | 18% | 15% | 21% | 15% | 14% | 10% | 8% | 15% | 15% | 34% |
| **Cortical GM (left)** | 7% | 8% | 12% | 7% | 19% | 15% | 10% | 8% | 8% | 12% | 11% | 18% |
| **Cortical GM (right)** | 6% | 6% | 15% | 7% | 14% | 10% | 11% | 6% | 6% | 12% | 13% | 12% |
| **Cortical GM (bilateral)** | 6% | 6% | 13% | 7% | 16% | 11% | 10% | 7% | 7% | 11% | 12% | 15% |
| **Cortical GM+ subcortical WM (left)** | 7% | 7% | 12% | 7% | 20% | 14% | 11% | 8% | 8% | 11% | 11% | 20% |
| **Cortical GM+ subcortical WM (right)** | 6% | 6% | 15% | 7% | 15% | 10% | 11% | 6% | 6% | 11% | 13% | 13% |
| **Cortical GM+ subcortical WM (bilateral)** | 6% | 6% | 13% | 7% | 17% | 11% | 10% | 7% | 6% | 11% | 12% | 16% |
| **Subcortical GM (left)** | 10% | 9% | 18% | 14% | 19% | 22% | 16% | 13% | 10% | 18% | 15% | 39% |
| **Subcortical GM (right)** | 15% | 15% | 20% | 16% | 22% | 24% | 16% | 14% | 15% | 20% | 15% | 30% |
| **Subcortical GM (bilateral)** | 12% | 11% | 18% | 15% | 19% | 22% | 15% | 13% | 11% | 16% | 14% | 34% |
| **Auditory subcortex WM** | 8% | 9% | 22% | 9% | 20% | 21% | 14% | 10% | 9% | 18% | 10% | 24% |
| **Auditory cortex GM** | 7% | 8% | 19% | 8% | 18% | 23% | 14% | 8% | 9% | 19% | 11% | 21% |
| **Auditory cortex/subcortex GM+WM** | 7% | 8% | 20% | 9% | 19% | 21% | 14% | 9% | 9% | 18% | 11% | 22% |
| **Occipital subcortex WM** | 12% | 12% | 26% | 12% | 22% | 17% | 13% | 12% | 7% | 20% | 14% | 15% |
| **Occipital cortex GM** | 11% | 12% | 23% | 10% | 18% | 17% | 14% | 12% | 7% | 16% | 14% | 16% |
| **Occipital cortex/subcortex GM+WM** | 11% | 12% | 24% | 11% | 19% | 17% | 13% | 12% | 6% | 18% | 14% | 16% |
| **Temporal subcortex WM** | 6% | 8% | 18% | 7% | 18% | 13% | 11% | 9% | 8% | 12% | 10% | 22% |
| **Temporal cortex/subcortex GM+WM** | 5% | 8% | 18% | 7% | 16% | 14% | 10% | 8% | 8% | 12% | 10% | 20% |
| **Frontal subcortex WM** | 8% | 7% | 11% | 10% | 23% | 16% | 18% | 9% | 8% | 15% | 16% | 20% |
| **Frontal cortex GM** | 8% | 7% | 12% | 9% | 20% | 16% | 18% | 8% | 9% | 15% | 15% | 18% |
| **Frontal cortex/subcortex GM+WM** | 8% | 7% | 11% | 9% | 21% | 15% | 18% | 9% | 8% | 15% | 15% | 19% |
| **Visual cortex GM** | 13% | 17% | 27% | 12% | 24% | 22% | 15% | 15% | 8% | 21% | 17% | 19% |
| **Visual cortex/subcortex GM+WM** | 13% | 16% | 29% | 12% | 25% | 21% | 15% | 14% | 7% | 21% | 16% | 16% |
| **Primary somatosensory cortex GM** | 8% | 10% | 18% | 9% | 29% | 15% | 20% | 10% | 9% | 15% | 16% | 19% |
| **Pallidum** | 14% | 12% | 31% | 24% | 31% | 38% | 20% | 15% | 19% | 31% | 26% | 41% |
| **Hippocampus** | 16% | 17% | 27% | 18% | 22% | 24% | 22% | 18% | 16% | 24% | 18% | 24% |
| **Corpus callosum** | 16% | 16% | 27% | 19% | 45% | 29% | 23% | 15% | 17% | 27% | 22% | 42% |

Supplementary Table 4: Inter-subject CVs of the concentration estimates per ROI displayed in Sup.Tbl. 3. As expected, higher SNR/concentration metabolites corresponded to the lowest CVs.

| **Supplementary Table 5 - MRSinMRS checklist** | |
| --- | --- |
| **Site (Name or Number)** | **Vienna HFMRC** |
| **1. Hardware** |  |
| **a. Field strength [T]** | 7 |
| **b. Manufacturer** | Siemens |
| **c. Model (software version if available)** | Magnetom 7T |
| **d. RF coils: nuclei (transmit/ receive), number of channels, type, body part** | 1H, 32 ch, head, Nova Medical |
| **e. Additional hardware** | N/A |
| **2. Acquisition** |  |
| **a. Pulse sequence** | FID-MRSI |
| **b. Volume of Interest (VOI) locations** | Cerebrum |
| **c. Nominal VOI size [cm^3^, mm^3^]** | 220×220×110 |
| **d. Repetition Time (TR), Echo Time (TE) [ms, s]** | 450 ms / 1.3 ms acquisition delay |
| **e. Total number of Excitations or acquisitions per spectrum** | N/A, spatial-spectral encoding |
| **In time series for kinetic studies** | N/A |
| **i.         Number of Averaged spectra (NA) per time-point** | N/A |
| **ii.       Averaging method (e.g. block-wise or moving average)** | N/A |
| **iii.      Total number of spectra (acquired / in time-series)** | N/A |
| **f. Additional sequence parameters (spectral width in Hz, number of spectral points, frequency offsets); If STEAM: Mixing Time TM; If MRSI: 2D or 3D, FOV in all directions, matrix size, acceleration factors, sampling method** | BW 2778 Hz, 1920 spectral points, MRSI: 3D, 220×220×133 mm³, 64×64×39, spatial-spectral encoding |
| **g. Water Suppression Method** | WET |
| **h. Shimming Method, reference peak, and thresholds for “acceptance of shim” chosen** | Standard shim + manual adjustment, water peak < 50 Hz |
| **i. Triggering or motion correction method (respiratory, peripheral, cardiac triggering, incl. device used and delays)** | N/A |
| **3. Data analysis methods and outputs** |  |
| **a. Analysis software** | LCModel 6.3-1 |
| **b. Processing steps deviating from quoted reference or product** | Internal water reference |
| **c. Output measure (e.g. absolute concentration, institutional units, ratio)** | Concentration estimate |
| **d. Quantification references and assumptions, fitting model assumptions** | Simulated in NMRScope-B, macromolecular background |
| **4. Data Quality** |  |
| **a. Reported variables (SNR, Linewidth (with reference peaks))** | SNR and linewidths not reported |
| **b. Data exclusion criteria** | tCr SNR <5; tCr FWHM >0.15 ppm; metabolite Cramér–Rao lower bounds (CRLB) >40 % |
| **c. Quality measures of postprocessing Model fitting (e.g. CRLB, goodness of fit, SD of residual)** | CRLBs <40% for all off NAA, tCr, tCho, and mIns |
| **d. Sample Spectrum** | See Fig.1 |

Supplementary Table 5: A summary of the MRSI method according to the MRSinMRS expert’s consensus proposed standard^93^.

Supplementary Imaging Data: An overview of concentration estimate maps and additional data for a range of volunteers is available in NIFTI and MINC formats at Zenodo at <https://doi.org/10.5281/zenodo.5006923>.
